# Transcriptional bursts and heterogeneity among cardiomyocytes in hypertrophic cardiomyopathy

**DOI:** 10.3389/fcvm.2022.987889

**Published:** 2022-08-23

**Authors:** Valentin Burkart, Kathrin Kowalski, David Aldag-Niebling, Julia Beck, Dirk Alexander Frick, Tim Holler, Ante Radocaj, Birgit Piep, Andre Zeug, Denise Hilfiker-Kleiner, Cristobal G. dos Remedios, Jolanda van der Velden, Judith Montag, Theresia Kraft

**Affiliations:** ^1^Institute for Molecular and Cell Physiology, Hannover Medical School, Hannover, Germany; ^2^Institute for Cellular Neurophysiology, Hannover Medical School, Hannover, Germany; ^3^Clinic of Cardiology and Angiology, Hannover Medical School, Hannover, Germany; ^4^Mechanosensory Biophysics Laboratory, Victor Chang Cardiac Research Institute, Darlinghurst, NSW, Australia; ^5^Department of Physiology, VU University Medical Center, Amsterdam, Netherlands

**Keywords:** hypertrophic cardiomyopathy, burst-like transcription, cell-to-cell allelic imbalance, contractile imbalance, cardiomyocyte heterogeneity

## Abstract

Transcriptional bursting is a common expression mode for most genes where independent transcription of alleles leads to different ratios of allelic mRNA from cell to cell. Here we investigated burst-like transcription and its consequences in cardiac tissue from Hypertrophic Cardiomyopathy (HCM) patients with heterozygous mutations in the sarcomeric proteins cardiac myosin binding protein C (cMyBP-C, *MYBPC3*) and cardiac troponin I (cTnI, *TNNI3*). Using fluorescence *in situ* hybridization (RNA-FISH) we found that both, *MYBPC3* and *TNNI3* are transcribed burst-like. Along with that, we show unequal allelic ratios of *TNNI3*-mRNA among single cardiomyocytes and unequally distributed wildtype cMyBP-C protein across tissue sections from heterozygous HCM-patients. The mutations led to opposing functional alterations, namely increasing (cMyBP-C_c.927−2A>G_) or decreasing (cTnI_R145W_) calcium sensitivity. Regardless, all patients revealed highly variable calcium-dependent force generation between individual cardiomyocytes, indicating contractile imbalance, which appears widespread in HCM-patients. Altogether, we provide strong evidence that burst-like transcription of sarcomeric genes can lead to an allelic mosaic among neighboring cardiomyocytes at mRNA and protein level. In HCM-patients, this presumably induces the observed contractile imbalance among individual cardiomyocytes and promotes HCM-development.

## Introduction

Burst-like transcription and cell-to-cell allelic imbalance has been described for a variety of genes in global mRNA-transcriptome analyses (1, 2). Bursts originate from stochastic binding and unbinding of the transcription initiation complex to the promotor of a respective gene and depend on biochemical attachment and detachment rates (3). This stochastic process leads to pulses of transcription (4). Most likely, for each allele of a gene binding and unbinding occurs independently. This can be concluded from different fractions of mRNA from each allele that were detected among different cells and in basal variability in mRNA and protein expression among cells from the same population (5, 6). If both alleles encode for the identical protein, heterogeneity of allelic transcription from cell to cell is most likely negligible. However, in heterozygous patients, where one allele encodes for a disease causing mutation, allelic imbalance may exacerbate disease phenotype (7).

A severe disease that is caused by heterozygous mutations in genes encoding for sarcomeric proteins in almost all mutation-positive patients is Hypertrophic Cardiomyopathy (HCM) (8, 9). HCM is characterized by asymmetric hypertrophy of the left ventricle, the interventricular septum, or both. The myocardium of HCM-patients often shows a marked cardiomyocyte and myofibrillar disarray and increased fibrosis (10). Approximately 80% of mutation-positive HCM-cases carry a mutation in one of two sarcomeric proteins, β-myosin heavy chain (β-MyHC, *MYH7*) and cardiac myosin-binding protein C (cMyBP-C, *MYBPC3*). Mutations in cardiac troponin T (cTnT, *TNNT2*) and cardiac troponin I (cTnI, *TNNI3*) account for another 10% of mutation-positive HCM-cases (11). Notably, HCM-mutations alter sarcomeric function and thereby affect force generation of cardiomyocytes. Most mutations in β-MyHC, cTnI and cTnT are missense mutations. Amino acid substitutions affect e.g., ATPase function of β-MyHC, acto-myosin binding kinetics, stiffness of myosin heads, or activation of the thin filament (12). Most mutations in cMyBP-C are truncation mutations, where premature termination codons lead to nonsense-mediated decay of mutated mRNA (13). This leads to haploinsufficiency, a reduction in functional cMyBP-C protein, which also affects force generation (14). Yet, it remains unclear how mutations in different genes with different primary effects on force generation, e.g., calcium-sensitization (hypercontractility) or calcium-desensitization (hypocontractility), can lead to the same HCM-phenotype.

We hypothesized that burst-like transcription of sarcomeric genes in HCM-patients with heterozygous mutations could induce imbalanced expression of mutated and wildtype (WT) alleles at mRNA and protein level among neighboring cardiomyocytes. In patients with missense mutations, this would lead to cardiomyocytes with variable fractions of mutant protein. Since mutations directly affect mechanical function of cardiomyocytes, such heterogeneity is expected to have functional consequences for the myocardial syncytium. Cardiomyocytes with a larger proportion of mutated protein may show more severely altered contraction as compared to cells with lower fractions. In patients with truncation mutations, cells would contain divergent amounts of WT-protein and may thereby show differently altered force generation. Thus, burst-like transcription of HCM-genes may well cause contractile imbalance from cell to cell independent of the primary mutation effect and could thereby provide an important mechanism that promotes disease development in HCM. The resulting mosaic-like, variable force generation within the myocardium over time could disrupt the cardiac syncytium and lead to cardiomyocyte disarray and other HCM characteristics (15–17).

To test our hypothesis we analyzed single cardiomyocytes from patients with cMyBP-C truncation mutations which show calcium sensitization (18) and from patients with a missense mutation in cTnI which leads to calcium desensitization (19). We show that both genes are transcribed in bursts alongside a large variability in mutant vs. WT cTnI-mRNA and in WT-cMyBP-C-protein from cell to cell. The mosaic-like distribution of WT or WT/mutant protein from cell to cell most likely underlies the variable force generation among individual cardiomyocytes that we observed for patients with both kinds of mutations. Together with previous observations on HCM-related missense mutations in β-MyHC (15–17), our finding of transcriptional and functional heterogeneity among cardiomyocytes from patients with mutations that cause different primary effects suggests a common pathomechanism in heterozygous HCM-patients.

## Methods

An expanded methods section is available in the Supplementary material.

### Patients and donors

The ethics committee of Hannover Medical School approved the study on anonymized human tissue and experiments were carried out in accordance with the given recommendations (No. 2276–2014). Written informed consent according to the Declaration of Helsinki (20) was given by all subjects. Left ventricular septum tissue from HCM-patients was obtained either during myectomy surgery, or after heart transplantation. All patients were diagnosed with hypertrophic obstructive cardiomyopathy (HOCM) as evident from increased septal thickness (>13 mm). Myocardial samples of non-transplanted donor hearts without any known cardiovascular condition were obtained from the Sydney Heart Bank (21). Detailed information on mutations and clinical characteristics are given in Supplementary Table 1.

### Visualization of aTS by smRNA-FISH

Cryosections (10–14 μm) from frozen left ventricular heart tissue were hybridized with fluorescently labeled sets of 20-mer oligonucleotides for intronic or exonic sequences of RNA. Custom designed (Stellaris^®^ Probe Designer) intronic pre-mRNA probe sets labeled with fluorophore Quasar 670 (LGC Biosearch Technologies) and exonic mRNA probe sets with fluorophore Quasar 570 (LGC Biosearch Technologies) were used (Supplementary Table 2). After hybridization, aTS were counted as spots with co-localization of both Quasar 570 and Quasar 670 fluorescence in cardiomyocyte nuclei.

### Absolute quantification of *TNNI3*- and *MYBPC3*-mRNA in single cardiomyocytes

Cardiomyocytes were isolated from left ventricular cryosections (5 μm) by laser-microdissection (LMD) with a LMD6 setup (Leica). Cardiomyocytes were identified by striation pattern and staining of intercalated discs with an anti-cadherin antibody. Cells were cut by laser and captured in PCR-tubes. Successful capture of cardiomyocytes was verified microscopically within the Leica LMD6. After reverse transcription of *TNNI3* or *MYBPC3*-mRNA, cDNA from single cells and from *in vitro* transcribed *TNNI3* or *MYBPC3*-mRNA in serial dilutions was pre-amplified and quantified by real-time PCR, using a QuantStudio™ 6 Flex System (Thermo Fisher). RNA copies per cell were calculated from serially diluted standard-RNA.

### Relative quantification of mutant to WT *TNNI3-*mRNA in single cardiomyocytes

*TNNI3-*mRNA from LMD-isolated cardiomyocytes was reverse transcribed on a custom-made micro-mixer. Nested PCR was performed after splitting the whole sample as technical replicate. Reconditioned PCR-products were subjected to allele-specific restriction analyses with *Mwo*I for *TNNI*3_c.433C>T_ and *Bbs*I for donor *TNNI*3_SNP_ (single nucleotide polymorphism rs3729841). Allele-specific band patterns on agarose gels were quantified densitometrically and relative allelic fractions were calculated from band intensities.

### Identification of cMyBP-C truncation fragments and cMyBP-C protein quantification

Tissue samples from donors and HCM-patients were ground in a cryo-mortar and re-suspended in sample buffer. Proteins were separated by polyacrylamide gel electrophoresis and transferred to a nitrocellulose membrane by western blotting. cMyBP-C and α-actinin were detected by incubation with antibodies against the N-terminus of cMyBP-C or against α-actinin, respectively.

### Immunofluorescence protein staining in cryosections

Cryosections (5 μm) from cMyBP-C_trunc_ patient and donor tissue were fixed in 4% paraformaldehyde and immunofluorescently co-stained against cMyBP-C, α-actinin and N-cadherin. As secondary antibodies, Alexa Fluor 488, Alexa Fluor 555 and Alexa Fluor 680 were used simultaneously. DAPI was used to stain nuclei. Cryosections were analyzed by epifluorescent microscopy (H84) or confocal microscopy (H36, H45 and H89).

### Force measurements

Cardiomyocyte force generation and cross-bridge kinetics were characterized after mechanical isolation of single cardiomyocytes from flash frozen myocardial tissue as previously described (15, 17). Briefly, isolated and permeabilized cardiomyocytes were attached to a cantilever and a force transducer and treated with protein phosphatase 1-α (PP1-α) and protein kinase A (PKA) to adjust phosphorylation levels. Force was measured at different calcium concentrations (pCa-values) from relaxing (pCa 9.0) to maximal activating (pCa 4.18) calcium concentrations.

### Immunofluorescence protein staining in individual cardiomyocytes

Individual cardiomyocytes from cMyBP-C_trunc_ patient and donor were co-stained after functional measurements for cMyBP-C and α-actinin by specific antibodies and respective secondary antibodies in relaxing solution. Fluorescence was determined by confocal microscopy in the center of the cell.

### Quantification of hypertrophy and fibrosis marker expression by real-time PCR

RNA was extracted from donor and patient cardiac tissue and reverse transcribed using random decamers in three independent experiments. Fibrosis and hypertrophy marker gene expression was analyzed in duplicates by real-time PCR relative to four reference genes. Primers are given in Supplementary Table 3.

### Mathematical model of *TNNI3*-expression

The previously published (17) mathematical simulation of gene expression in individual cardiomyocytes was adapted for *TNNI3* and compared to results from smRNA-FISH, qPCR and functional measurements in donor and *TNNI*3_c.433C>T_-patients.

### Statistical analysis

Values are presented as mean ± SD unless otherwise indicated. Groups were compared using Mann-Whitney *U* test and group variances were compared using Levene's test. In multi-group comparisons one-way analysis of variance (ANOVA) and appropriate *post-hoc* tests were applied. Significance for all tests was accepted when *p* < 0.05. Statistical analysis and linear correlation test (Pearson correlation coefficient) was performed using GraphPad Prism and R.

## Results

### Burst-like transcription of *TNNI3* and *MYBPC3*

Single cardiomyocytes from donors (H89, H108 and H113, cf. Supplementary Table 1) and HCM-patients with a missense mutation in cTnI (H146 and H147 *TNNI*3_c.433C>T_, cTnI_R145W_) (19) or truncation mutations in cMyBP-C (H84, *MYBPC*3_c.927−2A>G_; H45, *MYBPC*3_c.1458−6G>A_; H36, *MYBPC*3_c.2864_2865delCT_; all denominated as cMyBP-C_trunc_) were analyzed. To test whether *TNNI3* and *MYBPC3* are transcribed continuous or burst-like, we used single molecule RNA fluorescence *in situ* hybridization (smRNA-FISH) analysis as previously described (17). Burst-like vs. continuous transcription can be tested by visualization of actively transcribed alleles. A continuously expressed gene would show two active alleles (or the maximal number of alleles in polyploid cells), determined as active transcription sites (aTS) in all cells. Stochastic, burst-like and independent expression of the alleles would appear as cells without aTS and cells with different numbers of aTS in the same tissue. Active transcription sites (aTS) in nuclei contain pre-mRNA – consisting of intronic and exonic sequences – and spliced mRNA – consisting only of exonic sequences. Fluorescently labeled probe sets for intronic (Quasar 670) and exonic (Quasar 570) RNA were hybridized to cryosections from heart tissue. Co-localization of both probes sets indicated aTS. To restrict the analysis to cardiomyocytes exclusively, cells with striation patterns and/or specific cytoplasmic mRNA spots were examined.

High sensitivity and specificity of our RNA-FISH assays was assessed in short-term cultivated human pluripotent stem cell derived cardiomyocytes (hPSC-CMs) where all nuclei reveal aTS for *MYBPC3*, indicating essentially continuous transcription (Supplementary Figure 1A). This demonstrates that the sensitivity of our assay will allow us to distinguish continuous and burst-like transcription. For *TNNI3*, modulation of transcriptional activity indicated high sensitivity of this FISH-assay. Treatment with triiodothyronine (T_3_) led to a substantial reduction of nuclei with aTS (Supplementary Figure 1A), indicating that a sensitive analysis of transcriptional activity is possible with our assay. To test whether we can depict differences in transcriptional activity in cardiac tissue, we analyzed left ventricular tissue from a one year old child. We show that transcription of both genes was substantially increased as detected by more nuclei with aTS. Furthermore, reproducibility among individual experiments, absence of specific signals in RNase-treated cardiac tissue and in human skeletal muscle tissue for both, *MYBPC3* and *TNNI3* (Supplementary Figures 1B–D), supports the high specificity of our assays.

**Figure 1:**
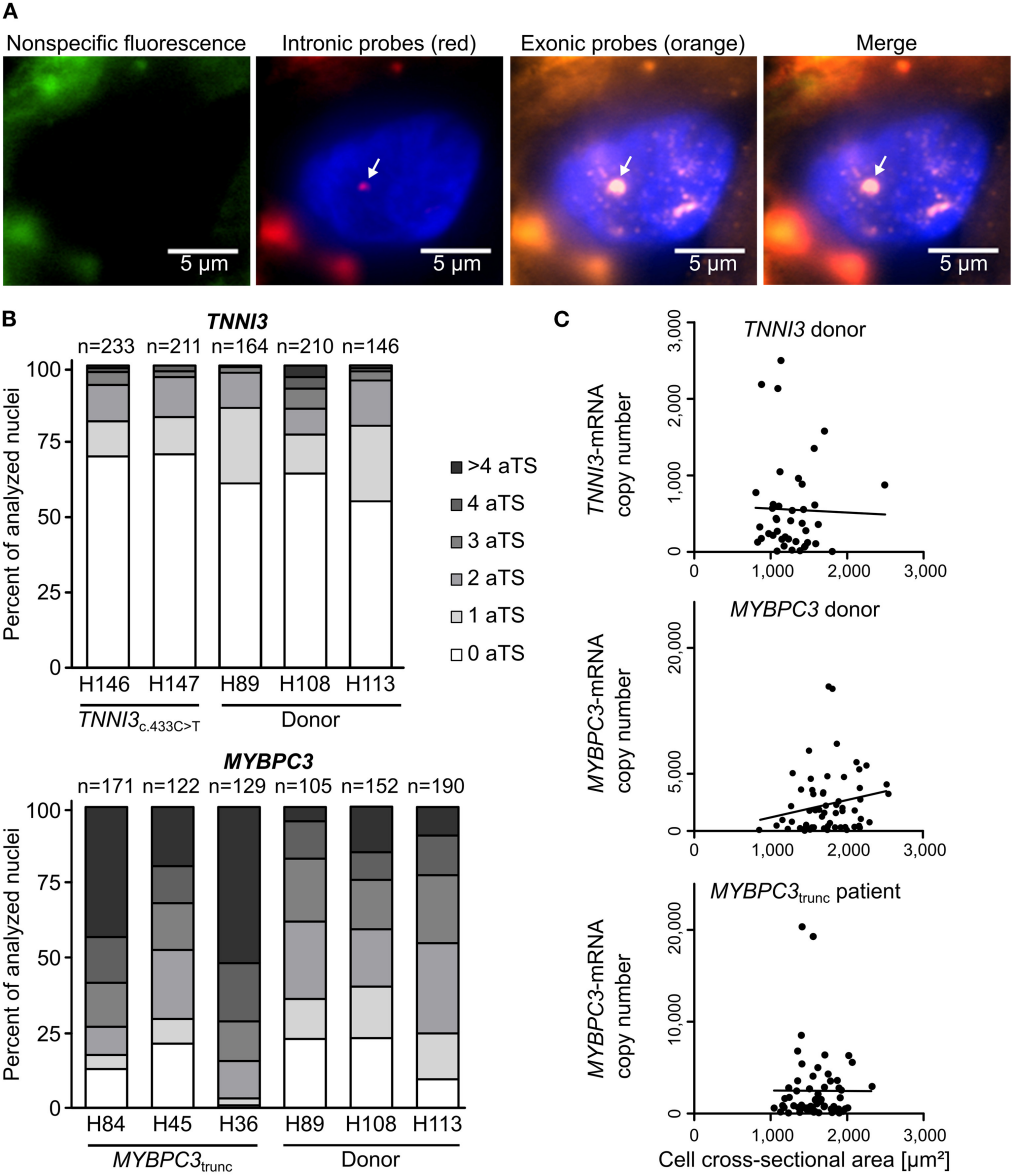
Independent, stochastic transcription of *TNNI3*- and *MYBPC3*-alleles. **(A)** Exonic and intronic mRNA of either *TNNI3* or *MYBPC3* were hybridized with fluorescently labeled DNA probes to visualize active transcription in cardiomyocyte nuclei in cryosections from left ventricular heart tissue. Representative cardiomyocyte nucleus with one active transcription site (aTS) for *MYBPC3* from the cMyBP-C_trunc_ patient. Left panel (green) non-specific fluorescence, middle panels intronic RNA (red) and exonic RNA (orange) and right panel merge. Intronic and exonic signals are shown merged with DAPI (blue) to stain the nucleus. One layer of the z-stack is shown. White arrow indicates aTS with co-localization of Quasar 570 and Quasar 670 fluorescence. The other fluorescent spots in the nucleus are only stained by exonic probes and presumably represent spliced mRNA. **(B)** Distribution of the numbers of aTS per nucleus in cardiomyocytes for *TNNI3* and *MYBPC3*. Percentage of nuclei with 0, 1, 2, 3, 4, and >4 aTS was plotted for different individuals. **(C)** *TNNI3*- or *MYBPC3*-mRNA copy number per cell plotted against the cell cross-sectional area. Linear correlation was tested using the Pearson correlation coefficient (*TNNI3*: *r* = −0.0263, *n* = 41, *p* = 0.870; *MYBPC3* donor: *r* = 0.1970, *n* = 63, *p* = 0.122 and *MYBPC*3_trunc_ patient: *r* = −0.0030, *n* = 61, *p* = 0.982).

To study the mode of transcription in adult human myocardium, smRNA-FISH was performed for *TNNI3* transcription in three donors and two cTnI_R145W_ patients and for *MYBPC3* transcription in three donors and three cMyBP-C_trunc_ patients. For donors and patients, myocardium from left ventricular wall and interventricular septum was analyzed (Supplementary Table 1). Figure 1A shows representative intronic and exonic signals in a nucleus from cMyBP-C_trunc_ heart tissue with one *MYBPC3*-aTS (further exemplary nuclei in Supplementary Figure 2). All patient and donor tissues contained cardiomyocyte nuclei without aTS and nuclei with one, two or more aTS of *TNNI3* or *MYBPC3*, respectively. Quantitative analysis revealed that most cardiomyocyte nuclei show no active *TNNI3*-transcription (Figure 1B). Donors showed 61, 64 and 55% of nuclei without aTS, cTnI_R145W_ patients had 70% and 71% nuclei without aTS. All individuals showed very few nuclei with more than two aTS. More active transcription was seen for *MYBPC3*, however, also here nuclei without aTS were detected; 23, 23, and 10% of nuclei in donors and 13, 21, and 1% in cMyBP-C_trunc_ patients were without aTS, respectively (Figure 1B).

In continuous transcription with constant rates for mRNA production, mRNA counts should follow a Poisson distribution, where mean and variance are equal. In contrast, burst-like transcription is associated with a non-Poisson distribution of mRNA per cell for a respective gene (22). To test this for *TNNI3* and *MYBPC3*, we quantified the total number of mRNA copies per cardiomyocyte from donor and the cMyBP-C_trunc_ patient tissue by real-time PCR. We determined a large heterogeneity of *TNNI3*-mRNA ranging from <20 to 2,499 copies per isolated cell with a mean of 552 molecules and a variance of 3.8^*^10^5^ (*n* = 41, SD = 613). A similar heterogeneity was found for *MYBPC3*-mRNA with copy numbers ranging from <200 to 15,776 per isolated cell with a mean of 2,937 molecules and a variance of 1.1^*^10^7^ (*n* = 63, SD = 3,329) in the donor. For the cMyBP-C_trunc_ patient a range from <200 to 20,338 with a mean of 2,486 molecules and a variance of 1.4^*^10^7^ (*n* = 61, SD = 3,769) was observed. In addition, mRNA copy numbers did not correlate with the cross-sectional area of isolated cardiomyocytes (Figure 1C), which would have been expected for cell-size specific transcription (23). Also, the frequency distribution of mRNA counts per cell showed no bimodality, which would be expected from continuous transcription from mono- and binucleated cells. This is in line with findings from human pluripotent stem cell derived cardiomyocytes (hPSC-CMs) where we show that the number of *MYH7*-mRNA molecules per cell varies significantly but does not correlate with the number of nuclei (Supplementary Figure 3). Similar results from single cell RNA-sequencing show that mono- and binucleated cells show comparable total mRNA levels for specific genes (24). Together, our findings strongly indicate burst-like transcription of *TNNI3-* and *MYBPC3*-alleles in donors and HCM-patients. Moreover, we found burst-like transcription in both, interventricular septum and left ventricular wall samples, in HCM-patients and donors.

### Unequal expression of mutated per WT *TNNI3*-mRNA in donor and patient cardiomyocytes

**Figure 2:**
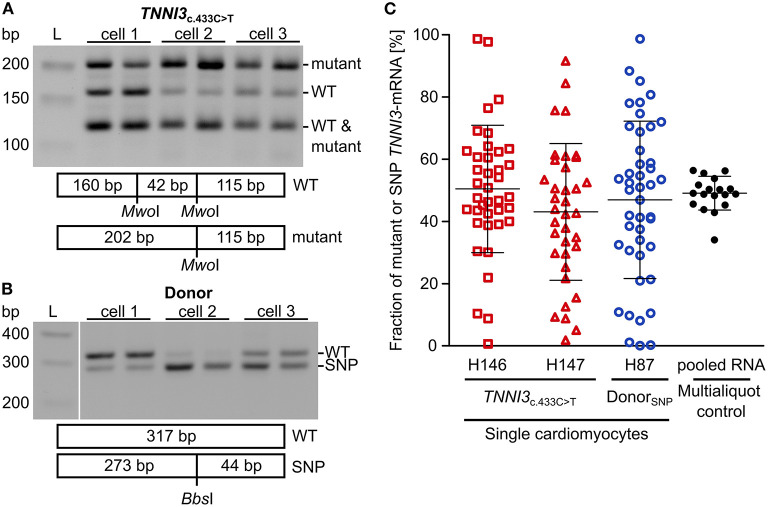
Allelic imbalance of *TNNI3* in cTnI_R145W_ patient and donor cardiomyocytes. Individual cTnI_R145W_ cardiomyocytes and donor cardiomyocytes were isolated by laser-microdissection from cryosections and reverse transcribed. cDNA was split in two technical replicates and subjected to nested PCR. **(A)** RT-PCR-products from single laser-microdissected cTnI_R145W_ cardiomyocytes were treated with *Mwo*I to generate allele-specific restriction fragments. Shown are three different cells in technical replicates. Expected fragment sizes are 202 bp for *TNNI*3_c.433C>T_-allele, 160 bp for WT-allele and 115 bp for both WT- and *TNNI*3_c.433C>T_-allele corresponding to 100% of the PCR-product. L indicates the DNA standard. **(B)** RT-PCR-products from donor cardiomyocytes with heterozygous SNP rs3729841 were treated with *Bbs*I to generate allele-specific restriction fragments. Shown are three different cells in technical replicates. Expected fragment sizes are 317 bp for WT-allele and 273 bp for SNP-allele. L indicates the DNA standard. **(C)** Fractions of mutant *TNNI3*-allele from single cTnI_R145W_ cardiomyocytes (two patients) and of SNP-allele from donor cardiomyocytes. Each dot represents the mean of two replicates from one cell. Multialiquot control, 18 individual PCR-analyses from pooled donor RNA with a concentration mimicking single cell level indicating the experimental error. Homogeneity of variance was tested by Levene's test, showing a significant difference between group variance [F(3, 133) = 6.72, p = 0.00029]. Lines indicate mean ± SD.

To examine whether burst-like transcription can induce allelic imbalance from cell to cell, the ratio of mutant per WT-mRNA in single cardiomyocytes from *TNNI*3_c.433C>T_ (cTnI_R145W_) patients and donors was analyzed. Single cardiomyocytes were isolated from cryosections *via* laser-microdissection and examined by RT-PCR in two technical replicates to detect dropout events. To quantify allelic expression, PCR-products were subjected to allele-specific restriction and resulting fragments were separated on agarose gels. PCR-linearity was validated with a set of plasmid mixtures of mutant and WT-allele (Supplementary Figure 4). Relative quantification in three independent experiments in duplicates showed, that both allelic templates were amplified and detected in the correct proportion with high accuracy (root mean square error = 5.3%). In Figure 2A representative restriction analyses of *TNNI3*-mRNA from individual *TNNI*3_c.433C>T_ cardiomyocytes are shown. The restriction enzyme *Mwo*I generates a 202 base pair (bp) mutation-specific fragment, a 160 bp WT-specific fragment and a 115 bp fragment from both alleles (Figure 2A). In this example, cell 1 showed comparable signal intensities for both mutant and WT-mRNA, whereas cells 2 and 3 had more intense signals for mutant specific fragments.

To analyze allelic expression in donor cardiomyocytes, we made use of a heterozygous non-pathogenic single nucleotide polymorphism (SNP rs3729841). Quantitative RT-PCR was performed with the same protocol as for patient cardiomyocytes and *Bbs*I was used for allele-specific restriction of PCR-products, generating a mutation-specific fragment of 273 bp and a WT-specific fragment of 317 bp. Figure 2B shows an exemplary gel analysis. Cell 1 shows a higher intensity for the WT-fragment, cell 2 for the SNP-fragment and cell 3 shows comparable intensities for both fragments.

To quantify allelic ratios in HCM-patients and donor, integrated optical densities (IOD) of allele-specific fragments were determined for each replicate and fractions of mutated or SNP- per WT-mRNA were calculated from respective IODs. Some cells showed a large difference between replicates, presumably due to technical limitations. Therefore, we performed a multialiquot control to determine the experimental scatter. Total RNA was extracted from five donor cryosections and diluted to single cell equivalent amount as determined by PCR-product intensity. We analyzed 18 aliquots using the same protocol as for single cardiomyocytes (Figure 2C). The mean of aliquots was 49.0% of SNP-mRNA with a standard deviation of 6.5%. Two-fold of this standard deviation (13.0%) was used as cut-off for the deviation between the two replicates that we generated upon quantification of transcripts from each single cardiomyocyte. In the final analyses, only cells where technical replicates from one cell differed <13.0% were included (Figure 2C).

We detected highly variable fractions of transcript from the mutated allele among single *TNNI*3_c.433C>T_ cardiomyocytes (Figure 2C, red symbols) and from the SNP-allele in donor cardiomyocytes (Figure 2C, blue symbols). Cells displayed the full range containing essentially only WT through variable mixtures of WT and mutant or SNP to essentially only mutant or SNP-mRNA. This variability was significantly larger than the experimental scatter and was similar for donor and *TNNI*3_c.433C>T_ cardiomyocytes (Figure 2C). It suggests similar heterogeneity of *TNNI3* allelic expression in donor cardiomyocytes and in HCM-patient cardiomyocytes. Importantly, the average fraction of mRNA from the two alleles (donor: 46.9% SNP-mRNA, patients: 50.4 and 43.0% mutant mRNA) was comparable to the mean fraction of mutant or SNP-mRNA from tissue sections (donor: 43.2% SNP-mRNA, patients: 46.0 and 54.4% mutant mRNA; Supplementary Table 5). This suggests that the analyzed single cardiomyocytes display a representative sample of cells from donor and patient tissue, respectively.

### Intra- and intercellular heterogeneous distribution of cMyBP-C protein in patient cardiomyocytes

**Figure 3:**
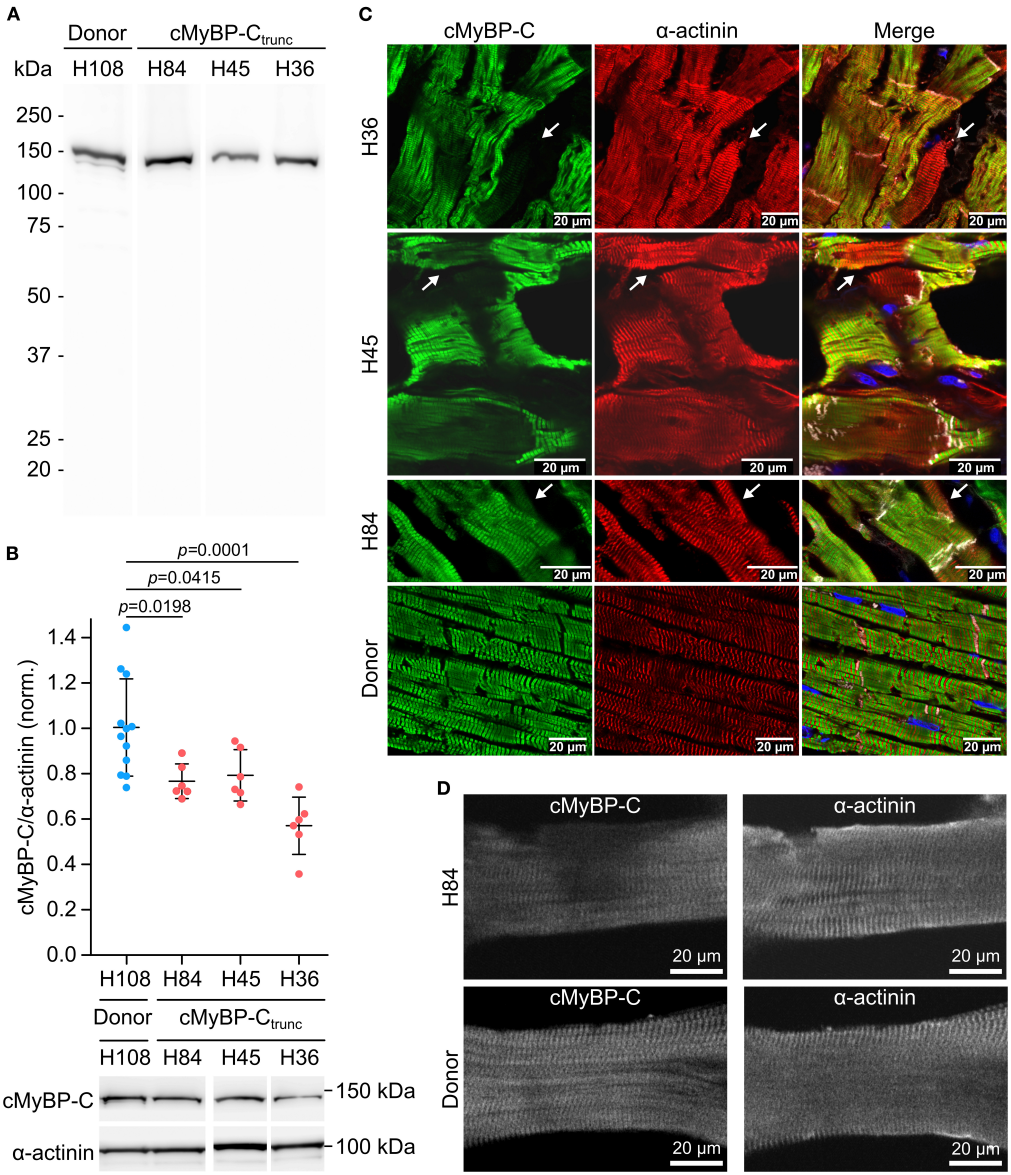
Unequal expression of cMyBP-C in cardiomyocytes of the cMyBP-C_trunc_ patient. **(A)** Western blot of one donor sample and three cMyBP-C_trunc_ patients H84 (*MYBPC*3_c.927−2A>G_), H45 (*MYBPC*3_c.1458−6G>A_) and H36 (*MYBPC*3_c.2864_2865delCT_) was incubated with an N-terminal cMyBP-C antibody. Detection of full-length WT-cMyBP-C (~140 kDa) and possible truncated cMyBP-C was performed with 5 s exposure time. **(B)** Relative protein amount of cMyBP-C was calculated from IOD of the cMyBP-C band divided by IOD of α-actinin for normalization. IOD of α-actinin band at 103 kDa was quantified as loading control. IOD ratio (cMyBP-C/α-actinin) from one donor sample (blue dots, n = 12 lanes) and compared to data from cMyBP-C_trunc_ patients (red dots, n = 6 lanes each). For comparison of patient results (H84, H45, H36) with donor (H108) reference one-way analysis of variance (ANOVA) and Dunnett's *post-hoc* test were performed. ANOVA yielded significant variation among groups [F(3, 26) = 10.17, p = 0.00013]. Mean±SD and p-values from Dunnett's test are indicated in the figure. **(C)** Cryosections (5 or 10 μm) from cMyBP-C_trunc_ patients and donor myocardium were stained with an N-terminus-specific antibody for cMyBP-C (left panels, green) to visualize cell-to-cell cMyBP-C distribution. Co-staining with an α-actinin antibody (middle panels, red) reveals sarcomeric Z-lines. Right panels, merge with N-cadherin (white) and DAPI (blue) staining. Images were either taken by confocal laser scanning microscopy (H36, *MYBPC*3_c.2864_2865delCT_ and H45, *MYBPC*3_c.1458−6G>A_) or by epifluorescent microscopy (H84, *MYBPC*3_c.927−2A>G_). White arrows indicate cells with substantially reduced cMyBP-C signals. **(D)** Confocal microscopy of single cardiomyocytes from cMyBP-C_trunc_ (c.927-2A>G, H84) patient or donor co-stained for cMyBP-C and α-actinin.

Mutations *MYBPC*3_c.927−2A>G_ (H84) and *MYBPC*3_c.1458−6G>A_ (H45) induce aberrant splicing and mutation *MYBPC*3_c.2864_2865delCT_ (H36) causes a frameshift; all mutations lead to a premature stop codon (25). Western blot analysis of cMyBP-C_trunc_ patient cardiac tissues showed no evidence for truncated cMyBP-C_trunc_ proteins (Figure 3A). Even with long exposure times of 120 seconds, only unspecific bands present in all analyzed samples could be detected (Supplementary Figure 5). Therefore, it seems unlikely that truncated protein was incorporated into the sarcomeres of cMyBP-C_trunc_ patients. Total full-length cMyBP-C per α-actinin was reduced to 80, 80, and 59%, respectively indicating haploinsufficiency (Figure 3B).

Burst-like transcription of mutant and WT-alleles may lead to different levels of WT-protein among cells. To test this, cryosections from patient's tissue were co-stained with antibodies against cMyBP-C and α-actinin. We observed a patchy distribution of cMyBP-C between individual neighboring cardiomyocytes in all three patients (Figure 3C, left column). Some cardiomyocytes showed no staining for cMyBP-C, whereas neighboring cardiomyocytes showed strong signals. In addition, we observed different intensities of cMyBP-C staining among cardiomyocytes. In contrast, α-actinin (Figure 3C, middle column) or β-MyHC (Supplementary Figure 6) as markers for sarcomere alignment showed a substantially more homogeneous pattern. In donor heart tissue, cMyBP-C distribution was homogenous (Figure 3C, lower row left panel) and similar to α-actinin (Figure 3C, lower row middle panel) and the β-MyHC pattern (Supplementary Figure 6). Z-stack and 3D views of cMyBP-C and α-actinin staining in patient tissue presented uneven cMyBP-C distribution also in z-dimension compared to donor tissue (Supplementary Videos 1–3). In summary, immunostaining of tissue from patients with three different haploinsufficiency-causing mutations reveals a substantial variability of WT-protein from cardiomyocyte to cardiomyocyte. Interestingly, as already evident from tissue staining, uneven distribution of cMyBP-C was also often found within patient cardiomyocytes (*MYBPC*3_c.927−2A>G_) when single, demembranated cardiomyocytes were co-stained for cMyBP-C and α-actinin (Figure 3D; Supplementary Figure 7). Whereas essentially all sarcomeres in individual cardiomyocytes could be stained for α-actinin, some sarcomeres or areas of these cardiomyocytes showed no signal for cMyBP-C (Figure 3D). In contrast, cardiomyocytes from donor heart tissue showed a considerably more even staining pattern for cMyBP-C and α-actinin over all sarcomeres (Figure 3D). Also, the confocal images of individual cardiomyocytes analyzed in functional measurements show lower levels and patchy distribution of cMyBP-C in patient cardiomyocytes compared to donor cells (Supplementary Figures 7A,B).

### Contractile imbalance among single cardiomyocytes

To test whether unequal expression of mutant and WT-alleles at mRNA and protein level also has consequences for cardiomyocyte contractile function or represents negligible fluctuations, calcium-dependent isometric force generation and cross-bridge kinetics of single, demembranated cardiomyocytes were examined. Cardiomyocytes isolated from flash frozen tissue of HCM-patients with cTnI_R145W_ and cMyBP-C mutation c.927-2A>G, respectively, were analyzed in comparison to donors as previously described (15). To adjust phosphorylation levels for patients and donor, cardiomyocytes were incubated with PP1-α and PKA prior to mechanical experiments (Supplementary Figure 8).

**Figure 4:**
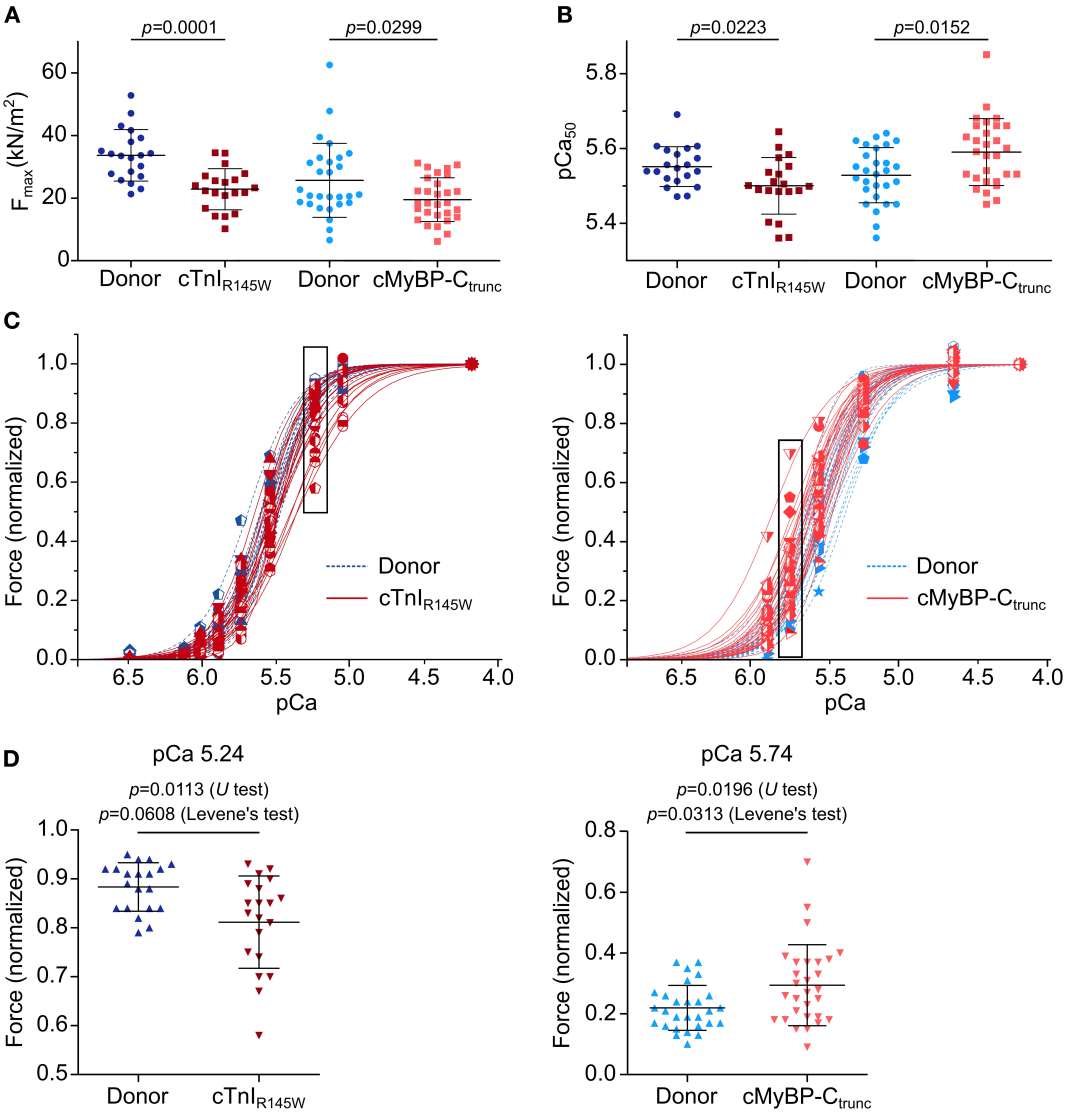
Contractile imbalance among cardiomyocytes from cMyBP-C_trunc_ and cTnI_R145W._ Individual cardiomyocytes from cTnI_R145W_ patient, cMyBP-C_trunc_ (c.927-2G>A) patient and respective donors were permeabilized and subjected to different calcium concentrations to measure calcium-dependent force generation. **(A)** Maximum force generation (pCa 4.18) and **(B)** pCa_50_ values of individual cTnI_R145W_ cardiomyocytes (n = 20), of cMyBP-C_trunc_ (n = 28) and of the corresponding donor cardiomyocytes (n = 20 and n = 29). pCa_50_ was derived from Hill-fits of normalized force-pCa relations. p-values of U test are indicated in the figure. **(C)** Force-pCa relations were plotted for individual cTnI_R145W_ cardiomyocytes, for cMyBP-C_trunc_ cardiomyocytes and the respective donor cardiomyocytes. Patient cardiomyocytes, red symbols and solid lines; donor cardiomyocytes, blue symbols and dashed lines. Force values in black boxes are plotted in **(D). (D)** Normalized forces of individual cardiomyocytes are shown for cTnI_R145W_ vs. the respective donor at pCa 5.24, and for cMyBP-C_trunc_ versus the respective donor at pCa 5.74. Each symbol represents an individual cardiomyocyte. Mean±SD and p-values for U-test and Levene's test are indicated in the figure.

On average, cardiomyocytes from the cTnI_R145W_ patient showed significant reduction of maximum isometric force by 32% and cardiomyocytes from the cMyBP-C_trunc_ patient by 24% compared to the respective donor (Figure 4A). This is in line with reports from the same patient with mutation cTnI_R145W_ and from patients with the same mutation in cMyBP-C (18, 19, 26). The rate constant of force redevelopment as measure for cross-bridge cycling kinetics was only slightly reduced (Supplementary Figure 9A). Interestingly, cardiomyocytes from the two patients showed opposite alterations in the calcium concentration needed for half maximal force generation (pCa_50_). pCa_50_ of the cTnI_R145W_ patient was significantly reduced to 5.50 ± 0.08 (mean ± SD) compared to the respective donor [pCa_50_ 5.55 ± 0.05 (mean ± SD)] indicating calcium desensitization (Figure 4B; Supplementary Figure 9B). In contrast, cMyBP-C_trunc_ cardiomyocytes had a significantly increased pCa_50_ of 5.59 ± 0.09 (mean ± SD) compared to the respective donor with a pCa_50_ of 5.53 ± 0.07 (mean ± SD), indicating calcium sensitization (Figure 4B; Supplementary Figure 9C). In addition, we observed that the Hill-fit did not characterize the force-pCa relationship of the cTnI_R145W_ cardiomyocytes well at higher calcium concentrations. Therefore, logit transformed force values were fitted with two linear functions (Supplementary Figure 9D). At low calcium concentrations, force generation of donor and cTnI_R145W_ cardiomyocytes was very similar, while at higher calcium concentrations (pCa <5.54) the slope for cTnI_R145W_ cardiomyocytes was significantly more shallow than for donor cardiomyocytes.

Analysis of calcium dependent force generation in individual cardiomyocytes from both patients revealed a marked variability that was considerably larger than that of donor cardiomyocytes. Some cTnI_R145W_ cardiomyocytes showed comparable force-pCa relations as donor cardiomyocytes, while others were significantly shifted to the right (Figure 4C). Variability in force generation between individual cTnI_R145W_ cardiomyocytes was strikingly visible at pCa values ≤5.54 (Figure 4D). The variance in force generation among the cTnI_R145W_ cardiomyocytes at pCa 5.54 was significantly increased by 3.6 fold as compared to donor cardiomyocytes (Levene's test *p* = 0.0608). cMyBP-C_trunc_ cardiomyocytes presented a similar large cell-to-cell variability, however, with a shift to the left (Figure 4C, right panel). The variance between the cMyBP-C_trunc_ cardiomyocytes was significantly increased by 3.2 fold compared to donor cardiomyocytes (Figure 4D, Levene's test *p* = 0.0313).

### Model calculations to connect transcriptional bursting to contractile imbalance of *TNNI3*

Since the different analyses could not be performed on one identical cardiomyocyte, we used a mathematical simulation to survey a potential direct link of the experimental findings in one cell. We modeled aTS distribution of *TNNI3*, allele-specific *TNNI3* transcription, allelic *TNNI3*-mRNA-fractions, number of total *TNNI3*-mRNA per cell and force generated at pCa 5.24 for 100,000,000 cells and compared them to experimental data from donors and from cTnI_R145W_ patients. For each individual simulated cell, relative force generation was consequence of the particular bursting and subsequent allelic mRNA and protein synthesis and degradation.

**Figure 5:**
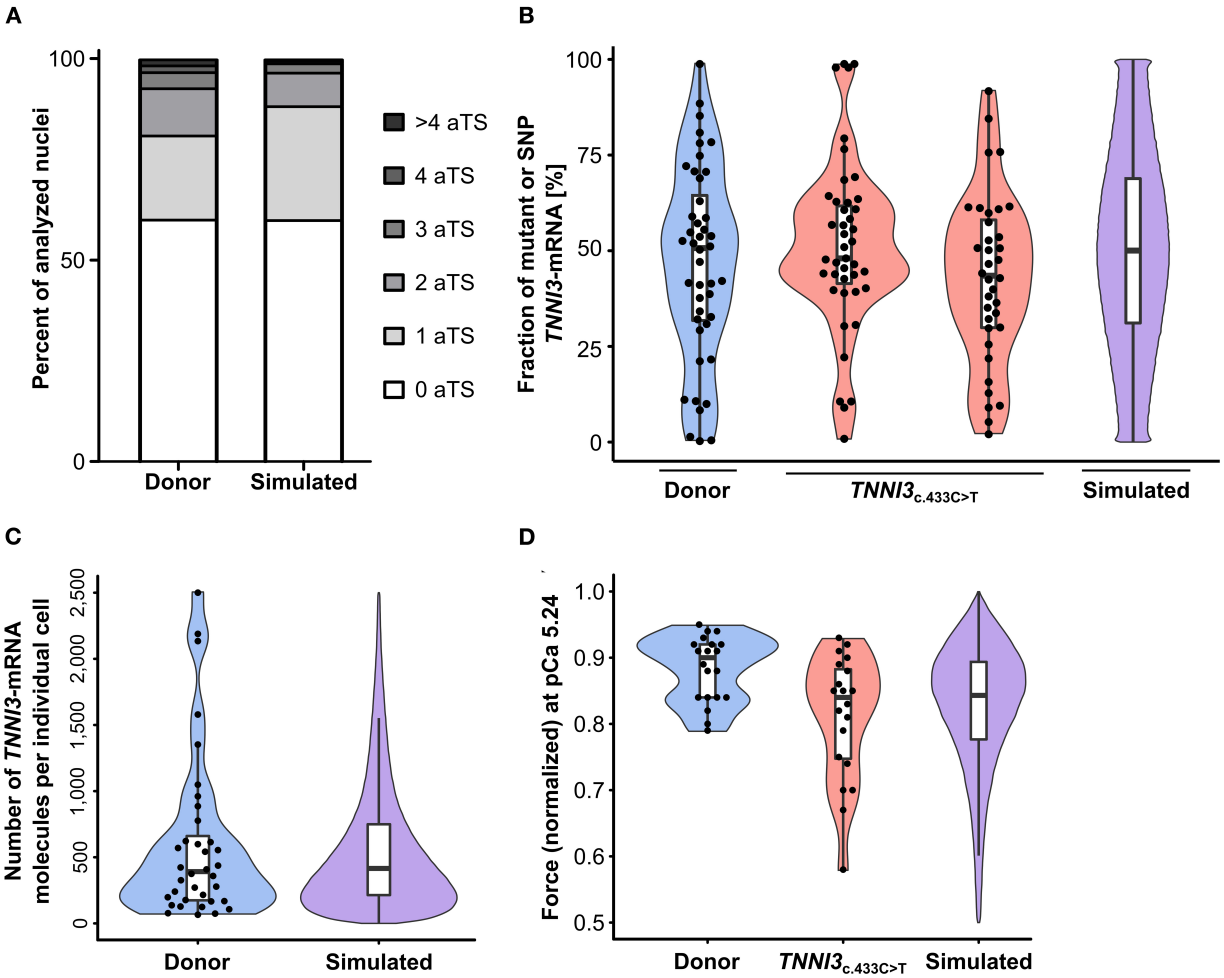
Experimental results from donor and from patients with *TNNI3*-mutations in comparison to simulation results from the model. **(A)** *TNNI3*-aTS distribution from three donors (H89, H108, and H113) compared to aTS distribution simulated by model. Percentage of nuclei with 0, 1, 2, 3, 4, and >4 aTS was plotted. **(B)** Percentage of *TNNI3-*SNP-allele transcripts from a donor and of *TNNI3-*mutant-allele transcripts from two cTnI_R145W_ patients (densitometric analysis of allele-specific restriction fragments), compared to the simulated distribution. Each data point represents the mean of replicates from one cell. Violin plots represent probability density of experimental data (blue and red) and probability density simulated by model (violet). Horizontal bar, median; white rectangle, interquartile range. **(C)** Total *TNNI3*-mRNA counts in donor cardiomyocytes. Violin plot probability density of the data (blue) compared to outcome of model simulation (violet). **(D)** Force values of individual cardiomyocytes for donor and for *TNNI*3_c.433C>T_ patient at pCa 5.24, normalized to respective maximum force, compared to simulation outcome. Dots represent individual cardiomyocytes. Violin plots, probability density of data (blue and red) and of model simulation (violet).

*TNNI3*-aTS distribution from all three donors was averaged and compared to the simulation, which reproduced the trend to low numbers of nuclei with aTS (Figure 5A). Simulation of *TNNI3*-mRNA allelic expression revealed a comparable imbalance from cell to cell as determined experimentally, ranging from almost only mutant allele *via* different fraction of both alleles to essentially only wildtype allele (Figure 5B). The simulated *TNNI3*-allele ratio showed a broader and smoother distribution compared to the experimental data. This is presumably due to smaller numbers of data points in the experimental data. We next modeled the total number of *TNNI3*-mRNA copy number per cell. The mean of 580 *TNNI3* copies/cell was comparable to experimental data (552 copies per cell). Furthermore, the simulation could reproduce the distribution of *TNNI3*-mRNA counts (Figure 5C). To test whether mRNA allelic imbalance may lead to unequal fractions of mutant and wildtype protein and subsequently to contractile imbalance we simulated force generation of single cardiomyocytes based on the respective calculated fraction of mutant per wildtype cTnI-protein. This was done based on the assumption that higher fractions of mutant protein produce a higher effect on force development in the cardiomyocyte. The resulting simulated distribution of force generation was similar to the distribution of force generation at pCa 5.24 in patient cardiomyocytes (Figure 5D).

Additionally, the model also predicts the effect of burst-like transcription in individual cardiomyocytes over time, since our analysis on human tissue provided a snapshot of expression during tissue extraction. The model shows that fractions of mRNA from each allele change over time, resulting in changing fractions of mutant per wildtype protein and consequently force generation (Supplementary Figure 10).

## Discussion

Evidence accumulates that burst-like transcription is a general expression mode for most genes (2, 22). Both alleles of a respective gene are switched on and off stochastically and independently from each other. This leads to unequal fractions of mRNA from the alleles among individual cells within a tissue (17, 27). In heterozygous patients with mutations that alter protein function and biomechanical properties of a cell, such imbalanced allelic expression may cause functional heterogeneity from cell to cell, which could exacerbate disease phenotype (7, 16, 28). A vast majority of HCM-patients are heterozygous. Mutations occur in several different proteins and alter force generation in cardiomyocytes. However, primary effects of HCM-mutations are highly divergent, e.g. often causing calcium-sensitization but also calcium-desensitization (12). In addition, missense mutations impose their effect by incorporation of mutant protein into the sarcomere, whereas truncation mutations mostly lead to a reduction of functional protein, so-called haploinsufficiency (13). To date, HCM pathomechanisms are not fully understood. If burst-like transcription is a principle feature of sarcomeric gene transcription, it could represent an important general mechanism that exacerbates disease development in HCM.

To test this, we chose patients with mutations in different sarcomeric genes that cause opposing primary effects on force generation and analyzed sarcomeric gene transcription, allelic mRNA or protein expression, and force generation in donors and HCM-patients. We provide evidence that burst-like transcription is a physiological expression mode of sarcomeric genes, which leads to unequal fractions of mRNA from both alleles among individual cardiomyocytes. It does not affect protein expression in donor controls, since both alleles encode for the identical protein. In HCM-patients, however, we show that it leads to highly heterogeneous protein expression among cardiomyocytes. Along with that, we measured a substantial contractile heterogeneity among cardiomyocytes from each HCM-patient compared to donor cardiomyocytes. We suppose that this functional variability triggers the increased expression of marker genes for hypertrophy and fibrosis, which we detected in the patients.

### Burst-like transcription is a physiological transcription pattern that causes heterogeneity in heterozygous HCM-patients

Our finding that the sarcomeric genes *MYBPC3* and *TNNI3* are transcribed in bursts in donors indicates that this is a physiological transcription mode of these genes. Since it is also found for *MYH7* in donors (Supplementary Figure 11), we provide evidence that three of the most commonly affected genes in HCM are principally transcribed in bursts. This leads to transcriptional noise (29), as we show by variable mRNA-counts among cardiomyocytes for each gene. This variability is in a comparable range in single cell transcriptomic studies for cardiomyocytes in humans and mice (30, 31). Thus, it likely represents natural fluctuation, which can be tolerated within the myocardium. The variability also exists for other sarcomeric genes such as *TNNT2, TMP1, MYL2, MYL3*, and *ACTC1* (30) indicating a general expression mode for sarcomeric, HCM-associated genes. Therefore, we assume that the mechanisms we detected for *MYBPC3, TNNI3*, and *MYH7* can be transferred to further HCM-genes.

Variability in absolute mRNA molecule numbers per cell for each gene is accompanied by an unequal expression of the two alleles among individual cells (5), as we can show here for *TNNI3*. However, in donors this does not cause differential functionality of cells, since both alleles code for an identical protein. Nonetheless, burst-like transcription is maintained upon HCM-development, as seen by only small differences in transcriptional activity between HCM-patients and donors. *cTnI*_R145W_ patients showed comparable *TNNI3* transcriptional activity compared to donors. HCM-patients with cMyBP-C_trunc_ mutations showed a modest increase in *MYBPC3* transcriptional activity, which is still compatible with burst-like transcription. In HCM-patients, transcriptional bursts lead to unequal ratios of mutant per wildtype mRNA among individual cardiomyocytes. Here we showed for *cTnI*_R145W_ that the average mutant vs. wildtype mRNA ratio is 50:50 but ranges from 0:100 to 100:0 in individual cells. We suppose that mRNA allelic imbalance translates to imbalance at protein level. To date it is not possible to perform allele-specific quantification of single amino acid exchanges at protein level. However, in cMyBP-C_trunc_ patients, where mutated mRNA is degraded, unequal expression would lead to an inhomogeneous distribution of WT-cMyBP-C among cardiomyocytes, which we demonstrated in tissue sections from myocardium of three cMyBP-C_trunc_ patients with different truncation mutations. Previous reports showing unequal cMyBP-C distribution in HCM-patient cardiomyocytes with the same (32) and other truncation mutations in cMyBP-C (33) strengthen our observation.

Some cMyBP-C_trunc_ patients may exhibit altered cMyBP-C protein degradation or compensatory upregulation of cMyBP-C, which might correct for degradation of the mutated allele. These patients show no reduction in total cMyBP-C (33, 34). Here, the burden of truncated proteins could trigger disease, either by excess protein production and degradation or by inefficient incorporation of truncated protein in the sarcomeres and disruption of its structure (34). We assume that such mechanisms will affect cells with different fractions of mutant protein to different degrees, which would also cause heterogeneity among cells. The finding that patients without haploinsufficiency show an uneven distribution of cMyBP-C among cardiomyocytes supports this (33). Therefore, restoration of total cMyBP-C at tissue level still retains variable levels from cell to cell. In the patient analyzed in this study, upregulation of *MYBPC3*-transcriptional activity did not lead to increased amounts of *MYBPC3*-mRNA. The reduction of cMyBP-C in the same patient indicates that the increased transcriptional activity could not restore cMyBP-C protein level.

Most interestingly, we also observed a marked uneven intracellular distribution of cMyBP-C in patient cardiomyocytes but not in donors which is in line with cMyBP-C stainings in tissue from HCM-patients shown by Theis and colleagues (33). In rat cardiomyocytes it has been reported that mRNA from several sarcomeric genes is transported from the nucleus to the sarcomeres and translated directly at the Z-disc (35). The mRNA transcribed from one allele during a particular burst may thus be translocated to sarcomeres within a distinct cellular area. Upon translation at the Z-disc, the protein might be incorporated in adjacent sarcomeres. In cMyBP-C_trunc_ patients, where mutant protein is not found, this possibly causes areas without functional cMyBP-C leading to patchy intracellular distribution (36). If mRNAs transcribed from each nucleus in binucleated cardiomyocytes are transported to different areas of the cell similarly to skeletal muscle cells (37), this could additionally contribute to intracellular heterogeneity.

### Contractile imbalance between individual cardiomyocytes from HCM-patients with mutations in cTnI and cMyBP-C

We tested our hypothesis that highly variable force generation among individual cardiomyocytes can occur irrespective of the primary effect of the mutation on force generation. For missense mutations, different fractions of mutant protein will shift the force-pCa relationship to a different extent in each cardiomyocyte. For truncation mutations, different levels of wildtype cMyBP-C per cardiomyocyte can have the same effect. The direction of changes differs with the initial effect of the mutation on acto-myosin interaction. Thus, we analyzed calcium-dependent force generation of individual cardiomyocytes with mutation cTnI_R145W_ that causes a calcium-desensitization (19), and of cardiomyocytes with truncation mutation *MYBPC*3_c.927−2A>G_ that causes calcium-sensitization (18, 38). For both mutations, we detected a substantial heterogeneity in force generation among the individual cardiomyocytes from the patients. Even though also donor cardiomyocytes showed some variability in force generation, which reflects the experimental scatter and intrinsic physiological differences among cells, variability among patient's cardiomyocytes was significantly larger than that of the donors.

Importantly, we often encountered cells – mostly from patient tissue – with a low structural integrity presumably due to disease-associated cardiomyocyte damage in which force generation over the full range of calcium concentrations could not be examined. Since such cells were mainly found in patient tissue, variability between individual cardiomyocytes in HCM-patients most likely is even larger than shown by our analysis. A low number of cardiomyocytes, which show a large shift in force generation, presumably reflects this.

### Possible consequences of burst-like transcription for the pathomechanism of HCM

We show here for the first time that three of the most commonly affected genes in HCM are transcribed burst-like in donors, thus under physiological conditions. This general transcription mode is maintained in HCM-patients. It will have no functional effect in healthy individuals since they express only WT-allele transcripts and proteins (28). However, if one allele encodes for a mutated protein, which alters biomechanical function of cardiomyocytes, burst-like transcription creates not just fluctuation of transcripts but likely attains clinical impact, as shown here for mutations in *TNNI3* and *MYBPC3*. The observed heterogeneous force generation among cardiomyocytes from HCM-myocardium with either of the mutations most likely results from allele transcription in bursts. It is found in patients with truncation mutations as well as in patients with mutations that cause a poison peptide effect.

This contractile imbalance could disrupt the cardiac syncytium and induce disarray of cardiomyocytes (16, 17, 28). Aberrant stretch can induce expression of atrial naturetic peptide, angiotensin II, endothelin I and transforming growth factor β (TGF-β) in isolated cardiomyocytes (39, 40) and TGF-β in an HCM mouse model (41) and thereby lead to hypertrophy and fibrosis (39–41). In line with this, we show upregulated expression of several marker genes for fibrosis and hypertrophy in HCM-patients as compared to donors (Supplementary Figure 12). Interestingly, the extent of upregulation differed largely among individual patients, putatively reflecting the large variability in disease development which is characteristic for HCM (12). Future studies will reveal whether upregulation of these genes is also heterogeneous from cell to cell. Overall, contractile imbalance may activate pro-hypertrophic and pro-fibrotic pathways and for many different mutations exacerbate the HCM phenotype by inducing cardiomyocyte disarray and interstitial fibrosis (16, 17, 28).

It should be noted that the mutation effects, reduction in functional cMyBP-C or incorporation of functionally altered proteins into the sarcomeres, provide the primary disease mechanism. Homozygous patients, which presumably do not exhibit functional heterogeneity but develop heart failure, indicate this. Interestingly, the identical mutation affects homozygous and heterozygous patients differently. Whereas homozygous patients developed dilated cardiomyopathy, heterozygous patients developed HCM (42). This indicates that next to the direct effect of the mutation, functional heterogeneity among cardiomyocytes most likely exacerbates HCM phenotype development. Furthermore, HCM-development can be influenced by environmental stress, lifestyle and comorbidities like coronary artery disease, obstructive sleep apnea and renal diseases (43) as well as polymorphisms in other genes (44). This gets apparent in a family where several members carry the cTnI mutation R145W. However not all of them developed HCM and some even developed RCM (45). Burst-like transcription could also contribute to differences in disease severity within a family if the kinetics of bursting and/or mRNA and protein turnover would differ among family members, thus increasing or reducing cell-to-cell heterogeneity.

### Limitations of the study

One limitation of our study is that we cannot perform all analyses in the identical cell. Even though fluorescent staining of sarcomeres indicated different levels of cMyBP-C, robust absolute quantification was not feasible using this approach. To meet this limitation, we set up a mathematical simulation and tested whether variable force generation from cell to cell could result from burst-like transcription. We used our previously described computational model (17) and adjusted its rate constants for *TNNI3*. The model calculations show that burst-like transcription of *TNNI3* results in variable counts of aTS in the cells, marked cell-to-cell allelic imbalance and heterogeneous numbers of *TNNI3*-mRNA copies per cell, similar to our experimental data. Using our mathematical model we have previously shown that increased ploidy in HCM-patients does not influence the outcome of allelic imbalance among cardiomyocytes (17). The model also showed that the cell-to-cell allelic imbalance in HCM-patients results in large differences in force generation of patient cardiomyocytes, comparable to our experimental data.

We performed our study on isolated cardiomyocytes. In tissue, sarcomere length-dependent force generation was suggested to provide a smoothing effect on physiologically different layers of cardiomyocytes (46). Increased stretching of weaker cardiomyocytes by neighboring cells would accordingly increase their force and could thereby counteract contractile heterogeneity in a physiological range. However, loss of cMyBP-C was shown to lead to aberrant stretch activation (47) and perturbation of length-dependent force generation has been reported to be common in HCM (19). This feedback-mechanism could therefore fail to equalize forces of cardiomyocytes in HCM-tissue. Nevertheless, in future studies it may be feasible to investigate force generation of individual cardiomyocytes in larger preparations of HCM-patient's cardiac tissue.

Cellular adaptions to different forces may also occur *via* phosphorylation of regulatory proteins, such as cTnI, cMyBP-C or the regulatory myosin light chain. However, these mechanisms mostly act globally on all cells, whereas bursts will lead to diverse force levels among cardiomyocytes, which also change over time, as shown by our mathematical model. This would require constant and specific adaptations of phosphorylation for each cell to counteract the altered function, which seems unlikely. In addition, for HCM-mutation cTnI_R145W_ it was shown that it may induce a structural state of cTnI similar to PKC induced phosphorylation of cTnI at threonine 143 (48), which may contribute to inadequate adaptions to altered force generation. On the other hand, differential phosphorylation among cardiomyocytes could also cause heterogeneity in force generation. Analysis of both, adaption to differential forces by phosphorylation and differential phosphorylation as cause of heterogeneity would require single cell analysis of protein phosphorylation, which is not possible to date. To minimize potential influences of differential phosphorylation in individual cells on our measurements, we adjusted phosphorylation in all cardiomyocytes by treatment with PP1-α and PKA. Thus, the detected heterogeneity is most likely not due to differential phosphorylation, however we cannot exclude possible effects of other kinases such as PKC on sarcomeric proteins.

## Conclusion

We addressed the question whether sarcomeric proteins cMyBP-C and cTnI are transcribed burst-like, and how this may affect expression of mutated and WT-proteins as well as function of HCM-patient's cardiomyocytes. Alterations of the force generating mechanism in cardiomyocytes due to the respective HCM-mutation are the primary cause of the disease. Yet, transcription in stochastic, independent bursts of each allele at least for the sarcomeric proteins we studied so far (*TNNI3, MYBPC3* and *MYH7*) most likely is the cause of the observed contractile imbalance among cardiomyocytes. This over time may well contribute substantially to development of cardiomyocyte disarray, fibrosis and hypertrophy and thus exacerbate disease phenotype. Our current study provides evidence that functional heterogeneity among patient cardiomyocytes occurs irrespective whether the direct effect of the mutation is calcium-sensitization or calcium-desensitization. Thus, it seems likely that also alteration of other parameters of cardiac contraction such as shortening velocity or relaxation lead to contractile imbalance. Since three of the most commonly affected genes in HCM are transcribed in bursts, it is quite likely that transcriptional bursting will occur for other HCM-genes as well and promote development of hallmarks of HCM.

## Data availability statement

The original contributions presented in the study are included in the article/Supplementary material, further inquiries can be directed to the corresponding authors.

## Ethics statement

The studies involving human participants were reviewed and approved by Ethikkommission der Medizinischen Hochschule Hannover. Written informed consent to participate in this study was provided by the participants' legal guardian/next of kin.

## Author contributions

TK and JM designed the research. VB, KK, DA-N, JB, DF, TH, AR, BP, and AZ performed the research. DH-K, CR, and JV contributed patient or donor tissue. VB, JM, and TK wrote the paper. All authors contributed to the article and approved the submitted version.

## Funding

This work was supported by the Deutsche Forschungsgemeinschaft [grant number KR1187/22-1 to TK] and the European Research Area Network on Cardiovascular Disease [grant “SCALE”, BMBF Number 01KL2007 to JM].

## Conflict of interest

The authors declare that the research was conducted in the absence of any commercial or financial relationships that could be construed as a potential conflict of interest.

## Publisher's note

All claims expressed in this article are solely those of the authors and do not necessarily represent those of their affiliated organizations, or those of the publisher, the editors and the reviewers. Any product that may be evaluated in this article, or claim that may be made by its manufacturer, is not guaranteed or endorsed by the publisher.
