# Supplementary material for: Transcriptional bursts and heterogeneity among cardiomyocytes in hypertrophic cardiomyopathy
Source: Front Cardiovasc Med. 2022 Aug 23;9:987889. doi: 10.3389/fcvm.2022.987889 (PMC9445301; doi:10.3389/fcvm.2022.987889)
Supplement: Supplementary file 1 [file Data_Sheet_1.docx]

***Supplementary data***

**This PDF file includes:**

Supplementary Tables I to V

Supplementary Figures 1 to 12

Description of supplementary video material for cMyBP-C distribution

Supplementary methods

**Supplementary Table I: Available clinical data from all patient and donor samples used throughout the manuscript**

| **Name** | **Mutation (pathogenic)** | **Age, y** | **Sex** | **Symptoms** | **Comments and SNP**^‡^ **(nonpathogenic)** | **Sydney heart bank code** |
| --- | --- | --- | --- | --- | --- | --- |
| H30 | *MYH7*_c.2167C>G_ | 55 | m | 16 mm ST^†^ | explant, LV |  |
| H36 | *MYBPC3*_c.2864_2865delCT_ | 16 | f | 80 mm Hg LVOT* and 20 mm ST | myectomy, IVS |  |
| H45 | *MYBPC3*_c.1458-6G>A_ | 63 | m | 13 mm Hg LVOT and 20 mm ST | myectomy, IVS |  |
| H84 | *MYBPC3*_c.927-2A>G_  rs397516082 | 45 | m | NYHAII-III and ICD^§^; later NYHAIV and heart transplantation | explant, LV |  |
| H146 | *TNNI3*_c.433C>T_  rs104894724 | 46 | m | 100 mm Hg LVOT and 23 mm ST | myectomy, IVS |  |
| H147 | *TNNI3*_c.433C>T_  rs104894724 | 66 | m | 100 mm Hg LVOT and 16 mm ST | myectomy, IVS |  |
| H87 | None, donor | 53 | f | n.d.^‖^ | explant, LV; excess inotropes on life support; *TNNI3*_c.537G>A_ rs3729841 | 4.021 |
| H89 | None, donor | 41 | f | n.d. | explant, LV | 3.073 |
| H97 | None, donor | 40 | m | n.d. | explant, IVS | 6.008 |
| H108 | None, donor | 48 | m | n.d. | explant, IVS | 6.004 |
| H109 | None, donor | 38 | f | n.d. | explant, IVS | 6.024 |
| H113 | None, donor | 48 | m | n.d. | explant, IVS, LV^$^ | 6.052 |
| H172 | None, donor | 1.2 | f | n.d. | explant, LV | 5.114 |

*LVOT, left ventricular outflow tract pressure gradient; ^†^ST, septum thickness; ^‡^SNP, single nucleotide polymorphism; ^§^ICD, implantable cardioverter-defibrillator; ^‖^n.d., no data; LV, left ventricle used for analysis; IVS, intraventricular septum used for analysis; ^$^ LV was used for total RNA quantification, further analysis performed with IVS

**Supplementary Table II: RNA-Fluorescence in situ hybridization probes**

| ***TNNI3*** | | ***MYBPC3*** | |
| --- | --- | --- | --- |
| **Exonic probes**  **(5‘->3‘)** | **Intronic probes**  **(5‘->3‘)** | **Exonic probes**  **(5‘->3‘)** | **Intronic probes**  **(5‘->3‘)** |
| ttgagactccccgaggac | agtctgactcgcaaaccc | catcctgagagacgtcacac | tttccatgtatgtggacgag |
| cgggtgaccttcagggtc | tcccctctgaacaagagg | aaagctgagactggcttctt | ctgtgagtctctgtgctaag |
| ttggagggtcagtgaggg | atcaccaccaagacccca | gcaatgactgcgtaagatcc | cacaaggaggggttaacctg |
| gaatggcaggaggcaggg | aaaactccgcccctgaag | tctatgaccttgaggtcgaa | tgggcattcttggctttttc |
| cgccatgctgagactcag | gtcaccaatccgagcatg | attgagagctgctgagcttg | tgtagcctgatttttccttg |
| tggtgcagggcgaggttc | cttcccatctatccctaa | catcacgaagaggccaatgg | ggtgtggagagggttaaccg |
| aggagcggcgtctgattg | ggtctcttcctttggata | cttgaaccacttgaccacag | ctgctttgtttgcctgagag |
| tggcataagcgcggtagt | cattcccatccaccaatc | gaacagatagaccttgctgg | gtgagggtagttaacccaag |
| tttcttggcgtgcggctc | caagacacccccagcaaa | ctcgtggacagtgagattga | tcaacccctcagtggagaag |
| gaggcggagatcttagat | ggatcatggagggggatt | cagcttcttctgaaaggctg | agcgatggggtgatgggaaa |
| gctttgcaatctgcagca | cagtactccccgctaaag | cattcttgagccatttgacc | gatccctggcttaaatgggg |
| tggagctgtcggcacaag | gtctccactgttccaagg | tcaaagatgtacttgctgcc | agaggaacattggtggcatt |
| cttttgcctctatgtcgt | cttctaaaccctccagtt | caatgagcactggctgatgg | ctcagcagttcaggagttaa |
| ctccgtgatgttcttggt | ccggggcttcaggataaa | ctttcacaaagagctccgtg | ctacagtcctttgcttggtg |
| ttctgagtcagatctgca | tccgggactagaaacctc | ctccgatacttcacactcaa | gagttgtgtactcttcccag |
| tgcctcgaaggtcaaaga | tagaggctgtactgctga | gaaccggtatttgaaggtct | cttctcttctctcagcaacc |
| cagggtgggccgcttaaa | gacagccatattggacgc | tcgttgatgatcaggtggtg | aagcttgtcaggagaactgg |
| gcagagatcctcactctc | ggtcctgaaggagtaggt | ctagtgcacagtgcatagtg | ttaggggcttccagaaagac |
| agcagcgcctgcatcatg | tgttgttgggggaaccaa | cttcttttcctgcacaatga | tcggcgtagtgtgagagtca |
| gttttccttctcggtgtc | tcacagttgtcctgggtc | tcatctgagacctcacattt | ggttgaagggtgtggagaac |
| tgttcttgcgccagtctc | ccctgagcatgttcacta | cgatgtgggacacctttatg | catcccataggtgaaaaccc |
| cattccactcagtgcatc | gcctgagctaatttacca | tcgtcaatggtcagtttgtg | tccactaactacagcccttg |
| aactttttcttgcggccc | aatcacaattccctggcc | gggcacaaagctgtagtcag | ctctttccacagacagatgc |
| cagtaggcaggaaggctc | cataacctggtccagcta | aagtcaatcttgacctccat | atctctgagtcttggtccag |
| agctttattcctcagggc | tggaactgaatccccctc | aaccacaatggtgtctggta | cacagtctcaacacctgttc |
|  | cccagtctaggcttctaa | cagacgtagcttatttccag | tagtgggaacaggtgtcctc |
|  | agtctcttcccggcttag | cagcagcttcttgtcaaaca | taaaacgagaggcctggacc |
|  | tccagtaccgaggcctta | acagggttcttcactgtgac | gaacaaaccaggaatcagtc |
|  | aactcctccatcctacac | tcgatgaccttgactgtgag | tggcacgtatgattaggtga |
|  | cttccatttcccgcacac | cctgaatcaggtcgaagttc | ttttgtccctgttacaagac |
|  | tcagcatcctctttcctg | ttcaccagtatcgatgtgtg | cttttggccactttaaggaa |
|  | tcaggcctagggttgttg | accttcttctgaatggtctg | atgccctagactctgaactg |
|  | tgcttctacctccaactc | cggatgaacaggatggtgtc | tctgagcaagcctggggaag |
|  | atttctgaggacccctta | tggtaagtgcctgaatgcac | cagaggagaggtcagagagg |
|  | cttcctgtagccctaatg | actccagagccacattaaga | tcttgtgactgcacaaaggg |
|  | cttcctgtctgccttatg | aatgctccaagacggtgaac | tctctgcactttttccctag |
|  | tgcacttcctatctttcc | attgccaatgatgagctctg | catacaccaagagagccctt |
|  | tccacttcctgtctcctt | gctgaagacgcggaagtagt | tgttccagaactaagggact |
|  | tcaagataatccctgcca | ctgtcactaaagccaaccat | gtactgtggcttttactttc |
|  | tagttctggagcacttcc | tggtctggggataaagacgg | acactcctggaagagagctt |
|  | ctaccctggatgcctaag | tatagttgggtggctcatag | atatttacctgttggagctg |
|  | gtctccagtctctcaaga | agcagagcatagcagtgtag | agggaccaatctgactacat |
|  | gaggttagggtctcttct | tgaaccaggaaatcttgggc | aaagatggaccctttgttgg |
|  | tggaggatggcgatgagt | ttgctgaacatgcggaagcg | agctttgagaaccaatcctt |
|  |  | ctttctaatctccagagtca | tttagctcctgctaacacag |
|  |  | tacatccaacagtagggagg | agaaaaaagctgcctgctgg |
|  |  | attgggaagacatagcaggc | ttgagacaaggcccagagag |
|  |  | tctgtgactgcacttatctt | tgtgggtatagagtgggtag |

**Supplementary Table III: Primer for TNNI3-mRNA allelic quantification, total mRNA quantification and relative quantification of fibrosis and hypertrophy markers**

| **Primer** | **Sequence 5’->3’** |
| --- | --- |
| *TNNI3* RT | CAGCTCAGAGAGAAGCTTTA |
| *TNNI3* F1 | CCTCGAGAAAATTGCAGCTGA |
| *TNNI3* R1 | GGCAGTAGGCAGGAAGGCTCA |
| *TNNI3* F2 | CGCGGAGCTGCAGGACTT |
| *TNNI3* R2 | CGATGTTCTTGCGCCAGTCT |
| *TNNI3* real-time F | CTCCAACTACCGCGCTTATG |
| *TNNI3* real-time R1 | GCAGAGTCTTCAGCTGCAATTT |
| *TNNI3* real-time R2 | CATCTGCAGAGATCCTCACT |
| *TNNI3 in vitro* transcription forward | gatcactaatacgactcactatagggCCTCCAACTACCGCGCTTAT |
| *TNNI3 in vitro* transcription reverse | GGAATAAAGCTTCTCTCTGAGCTG |
| *MYBPC3* RT | CAGTACTCCACGCTGTAGCC |
| *MYBPC3* real-time F1 | ACAGGTGACAGCGATGAGTG |
| *MYBPC3* real-time R1 | GGGACCGATAGGCATGAAGG |
| *MYBPC3* real-time F2 | AGGACCAGGTCAACCTCACA |
| *MYBPC3* real-time R2 | CTCTCCCACGTTGCTGATCTT |
| *MYBPC3* probe | AAGGTCATCGACGTGCCA |
| *COL1A1* P1F | GTCGATGGCTGCACGAGTCA |
| *COL1A1* P1R | GCGGGAGGTCTTGGTGGTTT |
| *FHL1* P3F | ACTGCGTGACTTGCCATGAGA |
| *FHL1* P3R | CCTCCAGATGTGATGGCCTTGT |
| *GAPDH* P2F | GGTCGGAGTCAACGGATTTGG |
| *GAPDH* P2R | TGCCATGGGTGGAATCATATTGG |
| *IGF1* P6F | GGTGGATGCTCTTCAGTTCGT |
| *IGF1* P6R | TACCCTGTGGGCTTGTTGAAATA |
| *MYH6* P3F | ACTCCTGCGGCCCAGATTCTT |
| *MYH6* P3R | GGCCTCTAGACGCTCCTTCTCT |
| *MYH7* P2F | TGGCCACACCAAGGTGTTCTT |
| *MYH7* P2R | CTCGGGACTGGGCCTGGATA |
| *NPPA* P2F | CCACCGTGAGCTTCCTCCTT |
| *NPPA* P2R | TCCAAATGGTCCAGCAAATTCTTG |
| *NPPB* P2F | TGCAAGGGTCTGGCTGCTTTG |
| *NPPB* P2R | CTCTTAATGCCGCCTCAGCACTTT |
| *PGK1* P3F | TGGAGCTCCTGGAAGGTAAAG |
| *PGK1* P3R | ATGCCAAGTGGAGATGCAGAA |
| *POLR2A* P3F | TCTGCCCGTAACCAGGATGAC |
| *POLR2A* P3R | CGCCGCAGCTGATTGTTGAT |
| *RPL32* P3F | AGTTCCTGGTCCACAACGTC |
| *RPL32* P3R | GTGACTCTGATGGCCAGTTG |
| *TGBF1* P4F | GCGACTCGCCAGAGTGGTTA |
| *TGBF1* P4R | GCCGGTAGTGAACCCGTTGAT |

**Supplementary Table IV: TNNI3- and MYBPC3-cDNA Sequence used for in vitro transcription**

| ***TNNI3*-cDNA Sequence used for *in vitro* transcription** |
| --- |
| GATCACTAATACGACTCACTATAGGGCTCCAACTACCGCGCTTATGCCACGGAGCCGCACGCCAAGAAAAAATCTAAGATCTCCGCCTCGAGAAAATTGCAGCTGAAGACTCTGCTGCTGCAGATTGCAAAGCAAGAGCTGGAGCGAGAGGCGGAGGAGCGGCGCGGAGAGAAGGGGCGCGCTCTGAGCACCCGCTGCCAGCCGCTGGAGTTGGCCGGGCTGGGCTTCGCGGAGCTGCAGGACTTGTGCCGACAGCTCCACGCCCGTGTGGACAAGGTGGATGAAGAGAGATACGACATAGAGGCAAAAGTCACCAAGAACATCACGGAGATTGCAGATCTGACTCAGAAGATCTTTGACCTTCGAGGCAAGTTTAAGCGGCCCACCCTGCGGAGAGTGAGGATCTCTGCAGATGCCATGATGCAGGCGCTGCTGGGGGCCCGGGCTAAGGAGTCCCTGGACCTGCGGGCCCACCTCAAGCAGGTGAAGAAGGAGGACACCGAGAAGGAAAACCGGGAGGTGGGAGACTGGCGCAAGAACATCGATGCACTGAGTGGAATGGAGGGCCGCAAGAAAAAGTTTGAGAGCTGAGCCTTCCTGCCTACTGCCCCTGCCCTGAGGAGGGCCCTGAGGAATAAAGCTTCTCTCTGAGCTGAAAAAAAAAAAAAAAAAAAAAAAAAA |
| ***MYBPC3*-cDNA Sequence used for *in vitro* transcription** |
| GATCACTAATACGACTCACTATAGGGTGATCTGGCAGAAGGCTATCACGCAGGGGAATAAGGCCCCAGCCAGGCCAGCCCCAGATGCCCCAGAGGACACAGGTGACAGCGATGAGTGGGTGTTTGACAAGAAGCTGCTGTGTGAGACCGAGGGCCGGGTCCGCGTGGAGACCACCAAGGACCGCAGCATCTTCACGGTCGAGGGGGCAGAGAAGGAAGATGAGGGCGTCTACACGGTCACAGTGAAGAACCCTGTGGGCGAGGACCAGGTCAACCTCACAGTCAAGGTCATCGACGTGCCAGACGCACCTGCGGCCCCCAAGATCAGCAACGTGGGAGAGGACTCCTGCACAGTACAGTGGGAGCCGCCTGCCTACGATGGCGGGCAGCCCATCCTGGGCTACATCCTGGAGCGCAAGAAGAAGAAGAGCTACCGGTGGATGCGGCTGAACTTCGACCTGATTCAGGAGCTGAGTCATGAAGCGCGGCGCATGATCGAGGGCGTGGTGTACGAGATGCGCGTCTACGCGGTCAACGCCATCGGCATGTCCAGGCCCAGCCCTGCCTCCCAGCCCTTCATGCCTATCGGTCCCCCCAGCGAACCCACCCACCTGGCAGTAGAGGACGTCTCTGACACCACGGTCTCCCTCAAGTGGCGGCCCCCAGAGCGCGTGGGAGCAGGAGGCCTGGATGGCTACAGCGTGGAGTACTGCCCAGAGGGCTGCTCAGAGTGGGTGGCTGCCCTGCAGGGGCTGACAGAGCACACATCGATACTGGTGAAGGACCTGCCCACGGGGGCCCGGCTGCTTTTCCGAGTGCGGGCACACAATATGGCAGGGCCTGGAGCCCCTGTTACCACCACGGAGCCGGTGACAGTGCAGGAGATCCTGCAACGGCCACGGCTTCAGCTGCCCAGGCACCTGCGCCAGACCATTCAGAAGAAGGTCGGGGAGCCTGTGAACCTTCTCATCCCTTTCCAGGGCAAG |

**Supplementary Table V: Overview of mean fractions of TNNI3-R145W-mRNA**

|  | Single cardiomyocytes | | | Bulk tissue samples | | |
| --- | --- | --- | --- | --- | --- | --- |
|  | Donor | Patient 1 (cTnI_R145W_) | Patient 2 (cTnI_R145W_) | Donor | Patient 1 (cTnI_R145W_) | Patient 2 (cTnI_R145W_) |
| mean fraction of mutant *TNNI3*-mRNA [%] | 46.9 | 50.4 | 43.0 | 43.2 | 46.0 | 54.4 |
| Standard deviation | 25.3 | 22.4 | 22.1 | 4.5 | 3.5 | 3.1 |
| Number of analyzed cells or sections | 44 | 26 | 35 | 12 | 5 | 4 |

**
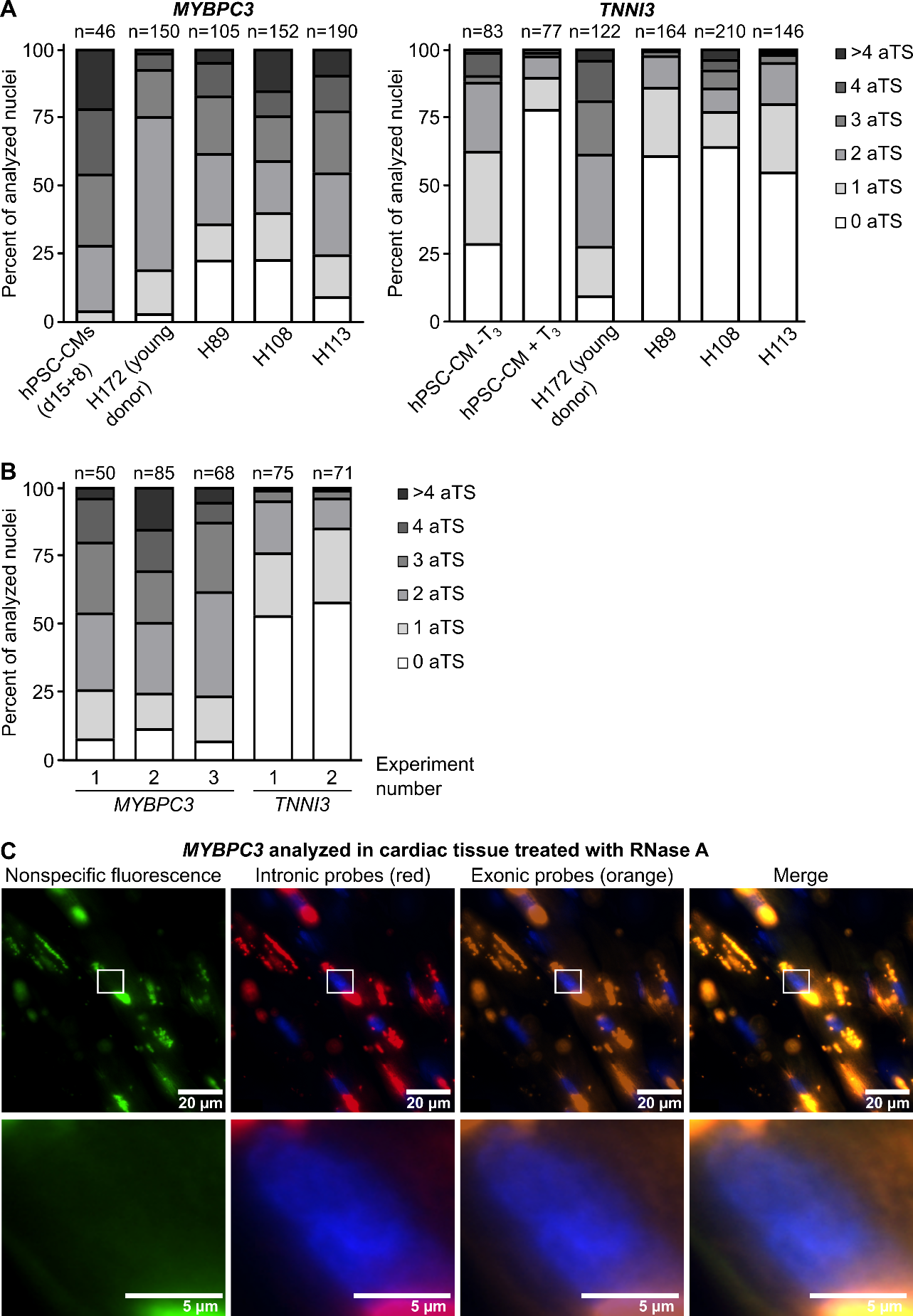
**

**Supplementary Figure 1**


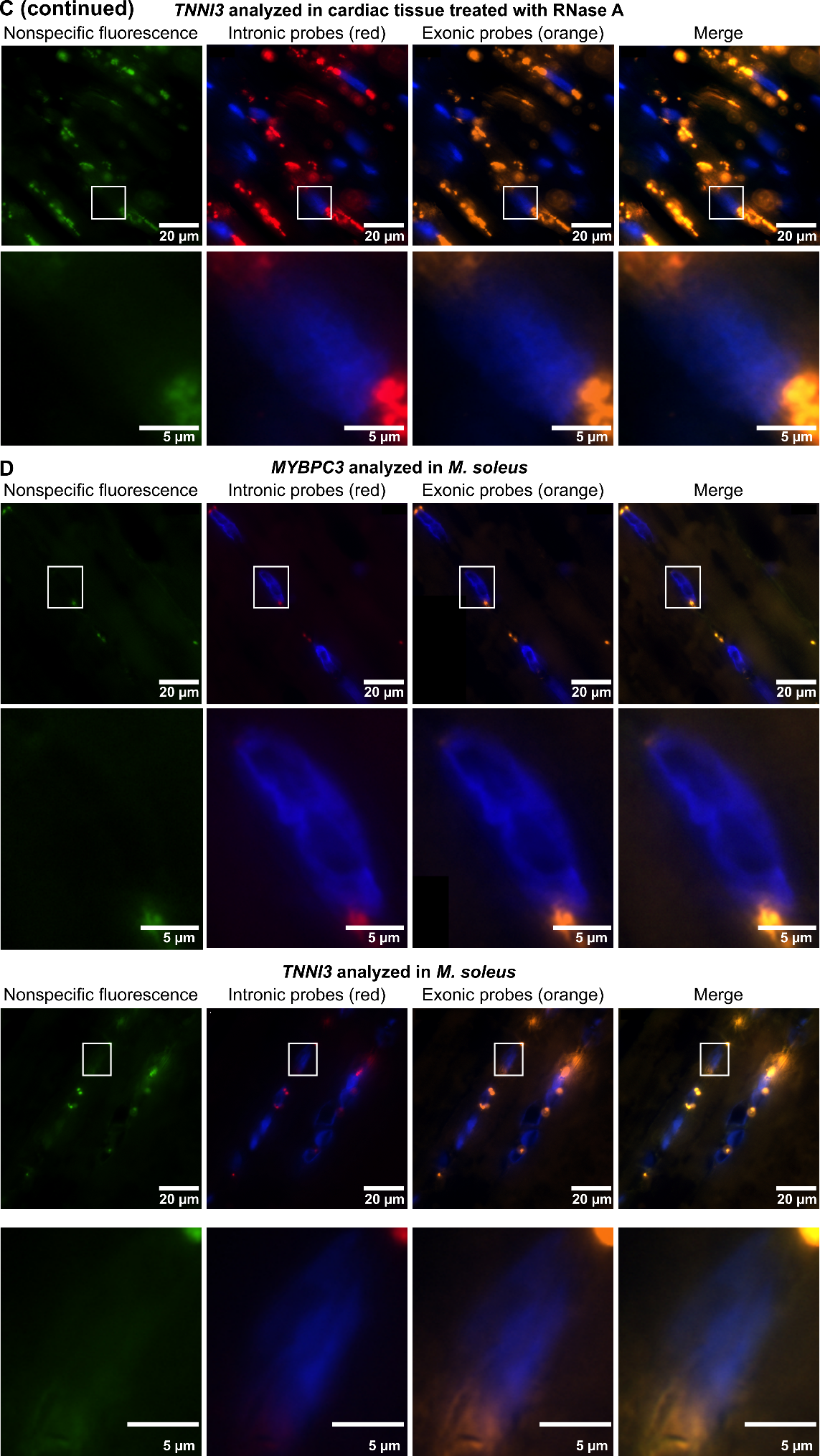


**Supplementary Figure 1: Sensitivity and specificity tests for RNA-FISH assays**

**(A)** To test for sensitivity of FISH-assays, samples with high expression levels for *MYBPC3* or *TNNI3* were analyzed. For *MYBPC3* (left panel), human pluripotent stem cell derived cardiomyocytes (hPSC-CMs) were fixed after 15 days differentiation and 8 days of cultivation as single cells on laminin-coated cover slips (d15+8) and subjected to RNA-FISH. In addition, left ventricular tissue sections from a one year old child (H172) were used for analysis. For *TNNI3* (right panel), hPSC-CMs (d12+8) were either cultivated for seven days with 200 ng/mL triiodothyronine (+T_3_) or left untreated (-T_3_, both d12+15) and used for RNA-FISH. In addition, cardiomyocyte nuclei of the one year old child were analyzed. Analysis of donor cardiomyocyte nuclei used for other experiments (H89, H113, and H108) are displayed for comparison. **(B)** Examples for reproducibility of RNA-FISH experiments are shown with three repeated experiments for *MYBPC3* and two repeated experiments for *TNNI3* with donor tissue of H113. aTS from at least 50 nuclei were counted per experiment and the resulting numbers showed only minor differences between the individual experiments. To analyze specificity, **(C)** cardiac tissue was treated with RNase A or **(D)** *M. soleus* muscle samples were subjected to FISH-analysis for *MYBPC3* (upper panels) or *TNNI3* (lower panels). Representative overviews show that no unspecific binding of the probe sets is detected. Representative nuclei from the same overview (marked with white rectangle) are shown enlarged for better visualization. Autofluorescence was detected in the GFP-channel as unspecific signals.


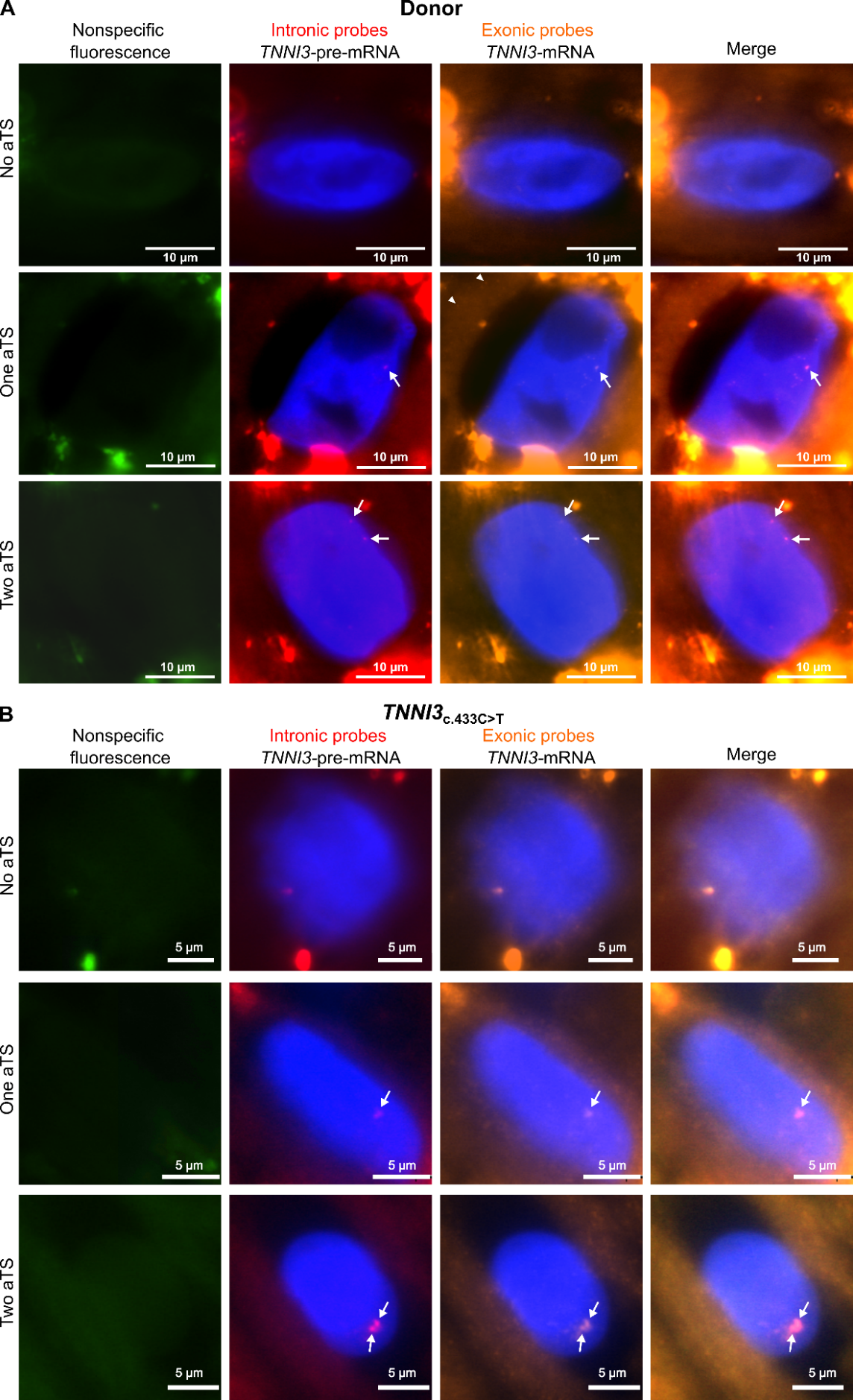


**Supplementary Figure 2**


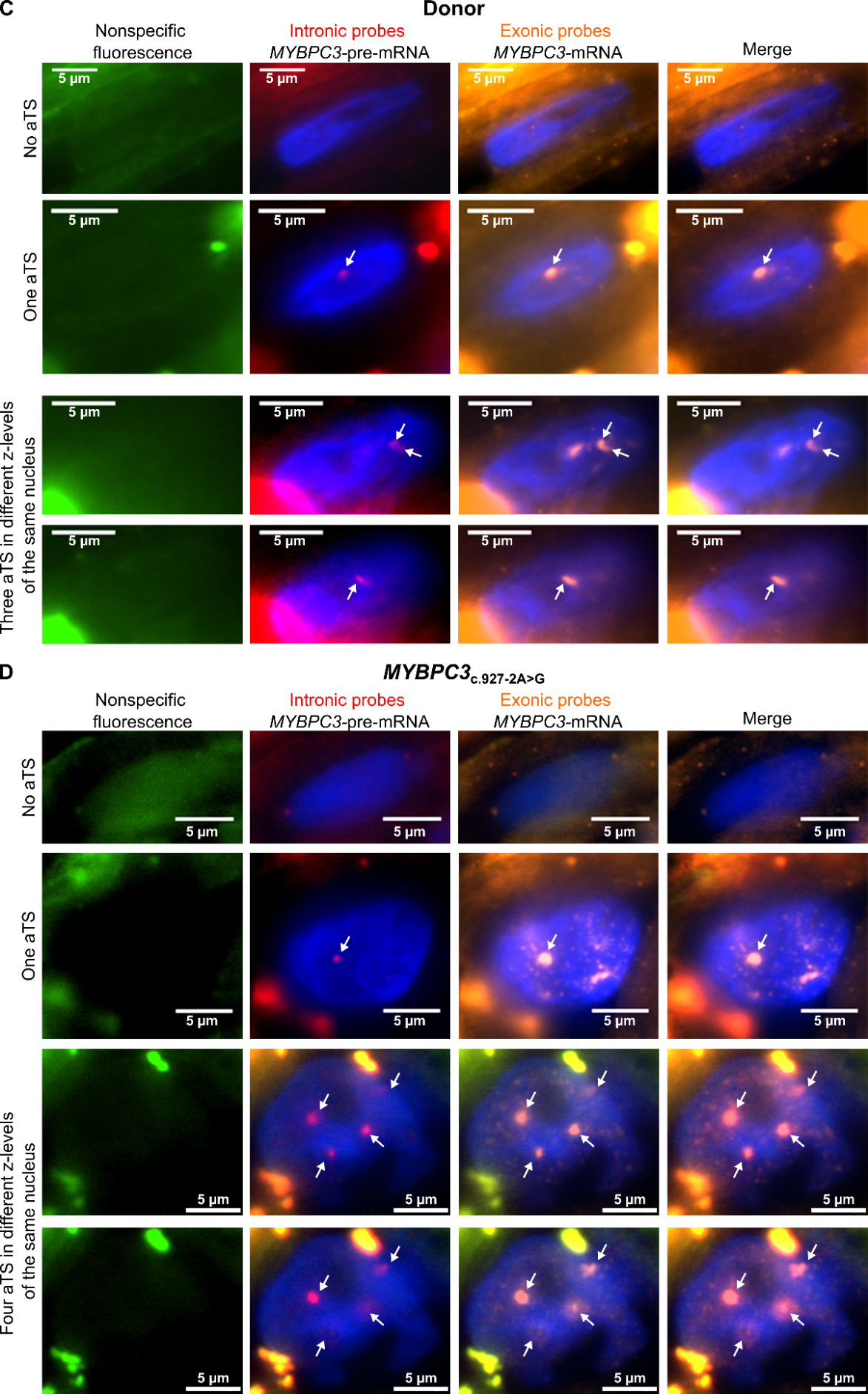


**Supplementary Figure 2**

**Supplementary Figure 2: Examples of RNA-FISH for TNNI3- and MYBPC3-aTS in patient and donor cardiomyocytes**

Cryosections (10-14 µm thickness) of left ventricular heart tissue were hybridized with fluorescently labeled DNA probes against exonic and intronic RNA from *TNNI3* or *MYBPC3*, respectively, to visualize active transcription in cardiomyocyte nuclei. Each panel shows one nucleus in different channels; first column: nonspecific fluorescence (green), second column: intronic transcript signals (red), third column: exonic transcript signals (orange), fourth column merge of intronic, exonic and DAPI (blue) fluorescence. Co-localization of intronic and exonic signals in the nucleus indicate active transcription sites (aTS), marked by white arrows. Small orange dots outside the nucleus (marked with white triangles) indicate cytoplasmic mRNA molecules (exonic sequence only). **(A)** Representative nuclei of donor cardiomyocytes with and without aTS for *TNNI3*. First row, nucleus without aTS; second row nucleus with one aTS; third row, nucleus with two aTS. **(B)** Representative nuclei of cTnI_R145W_ cardiomyocytes with and without aTS for *TNNI3*. First row, nucleus without aTS; second row nucleus with one aTS; third row, nucleus with two aTS. **(C)** Representative nuclei of donor cardiomyocytes with and without *MYBPC3-*aTS. First row, nucleus without aTS; second row, nucleus with one aTS; third and fourth row, same nucleus at two different z-levels presenting a total of three aTS in different locations. **(D)** Representative nuclei of cMyBP-C_trunc_ cardiomyocytes with and without *MYBPC3-*aTS. First row, nucleus without aTS; second row, nucleus with one aTS; third and fourth row, nucleus with four aTS in two different z-levels. The finding of cells without aTS, cells with one aTS and cells with two or more aTS for donors, *TNNI3*_c.433C>T_ patient, and *MYBPC3*_c.927A>G_ patient suggests burst-like transcription for both genes in all individuals.

*
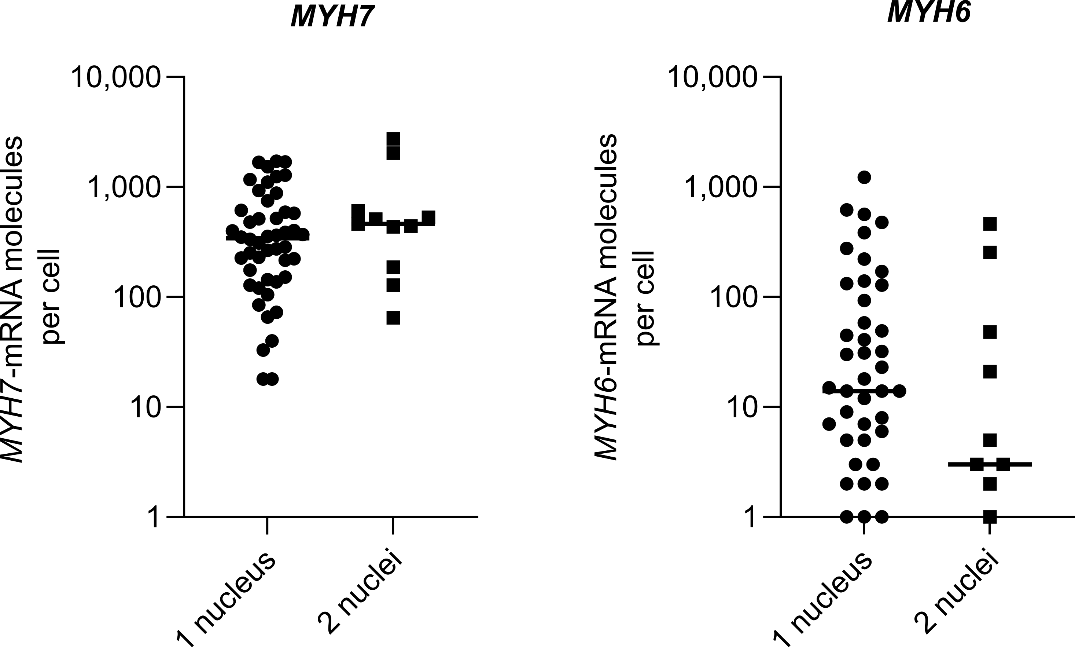
*

**Supplementary Figure 3: RNA-molecules per cell in relation to number of nuclei**

hPSC-CMs were cultivated for d14+35-41 days on laminin-coated cover slips, fixed and subjected to RNA-FISH analysis. Cytoplasmatic RNA-molecules for *MYH7* (left panel) or *MYH6* (right panel) were counted per cell and plotted according to the number of nuclei within the respective cell. Even though fewer cells with two nuclei were detected, range of mRNA molecules per cell was comparable in both groups for each gene. Additionally, comparable percentage of cells did not show *MYH6*-mRNA, namely 15% of cells with one nucleus and 18% of cells with two nuclei. This indicates that the number of mRNA-molecules per cell is independent of the number of nuclei in the individual cell.


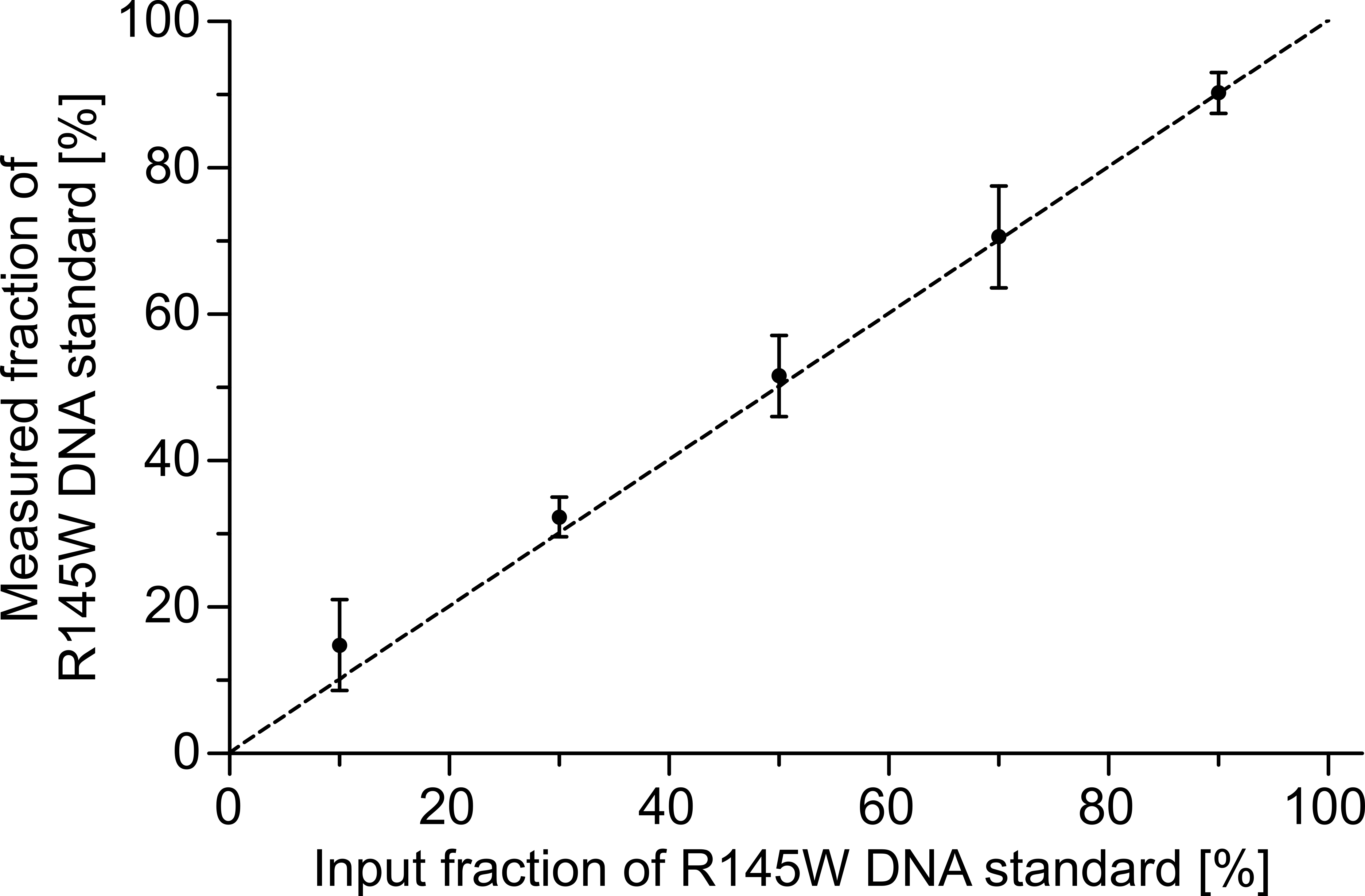


**Supplementary Figure 4: Control of linearity**

Analysis of a set of defined mixtures of mutant *TNNI3*_c.433C>T_ (cTnI_R145W_) and WT *TNNI3* synthetic plasmids. Dashed line represents the expected data if input and measured values were identical. Dots show the experimentally determined percentage of mutated templates plotted against the percentage of mutated templates inserted into the mixtures. Three independent experiments were performed in duplicates and the measured fraction of mutated DNA (%) was determined.


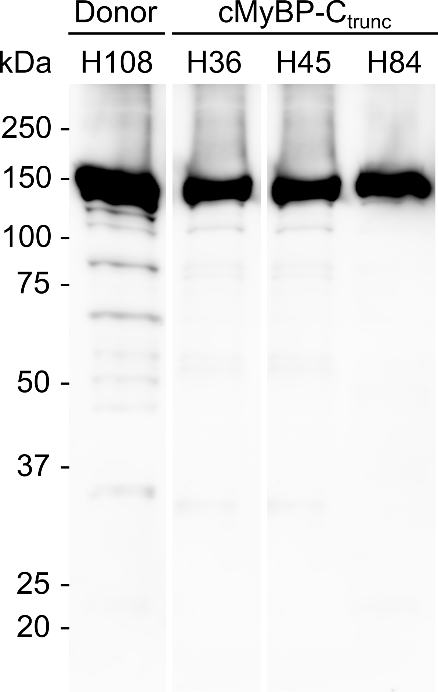


**Supplementary Figure 5: Western blot analysis of truncated cMyBP-C fragments at longer exposure times**

The same western blot as shown in Figure 3 is presented here with longer (120 seconds) exposure time. Western blot of one donor sample and three cMyBP-C_trunc_ patients H36, H45 and H84 (c.2864_2865delCT, c.1458-6G>A and c.927-2A>G) was incubated with an N-terminal cMyBP-C antibody. Detection of full-length WT-cMyBP-C (~140 kDa) and possible truncated cMyBP-C was performed with 120 seconds exposure time.


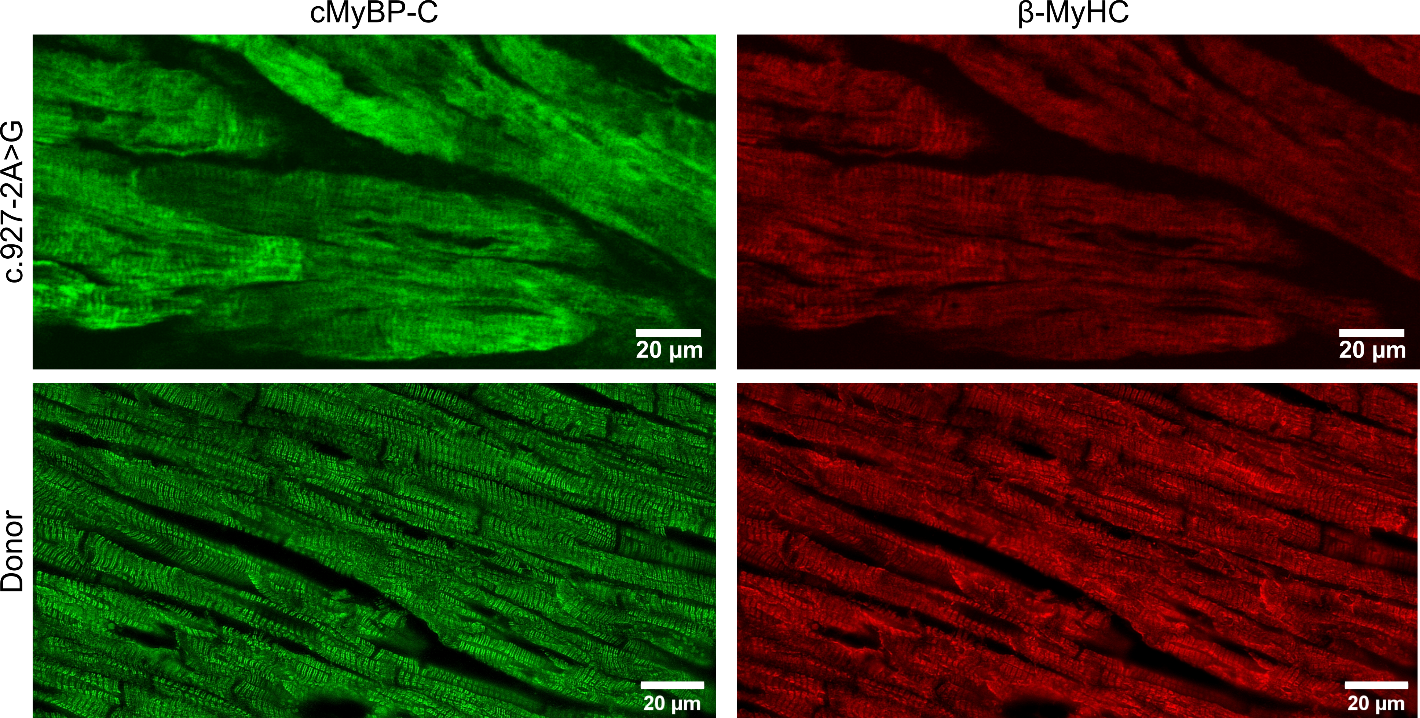


**Supplementary Figure 6: Co-immunofluorescence staining of cMyBP-C and β‑MyHC in cMyBP-C_trunc_ patient and donor myocardium**

Cryosections (5 µm) from cMyBP‑C_trunc_ patient and donor myocardium were stained with an N-terminus-specific antibody for cMyBP‑C (left panels, green) to visualize cell-to-cell cMyBP-C distribution. Co-staining with a β-MyHC antibody (right panels, red) reveals sarcomeric A-Bands. Images were analyzed by confocal laser scanning microscopy.

***
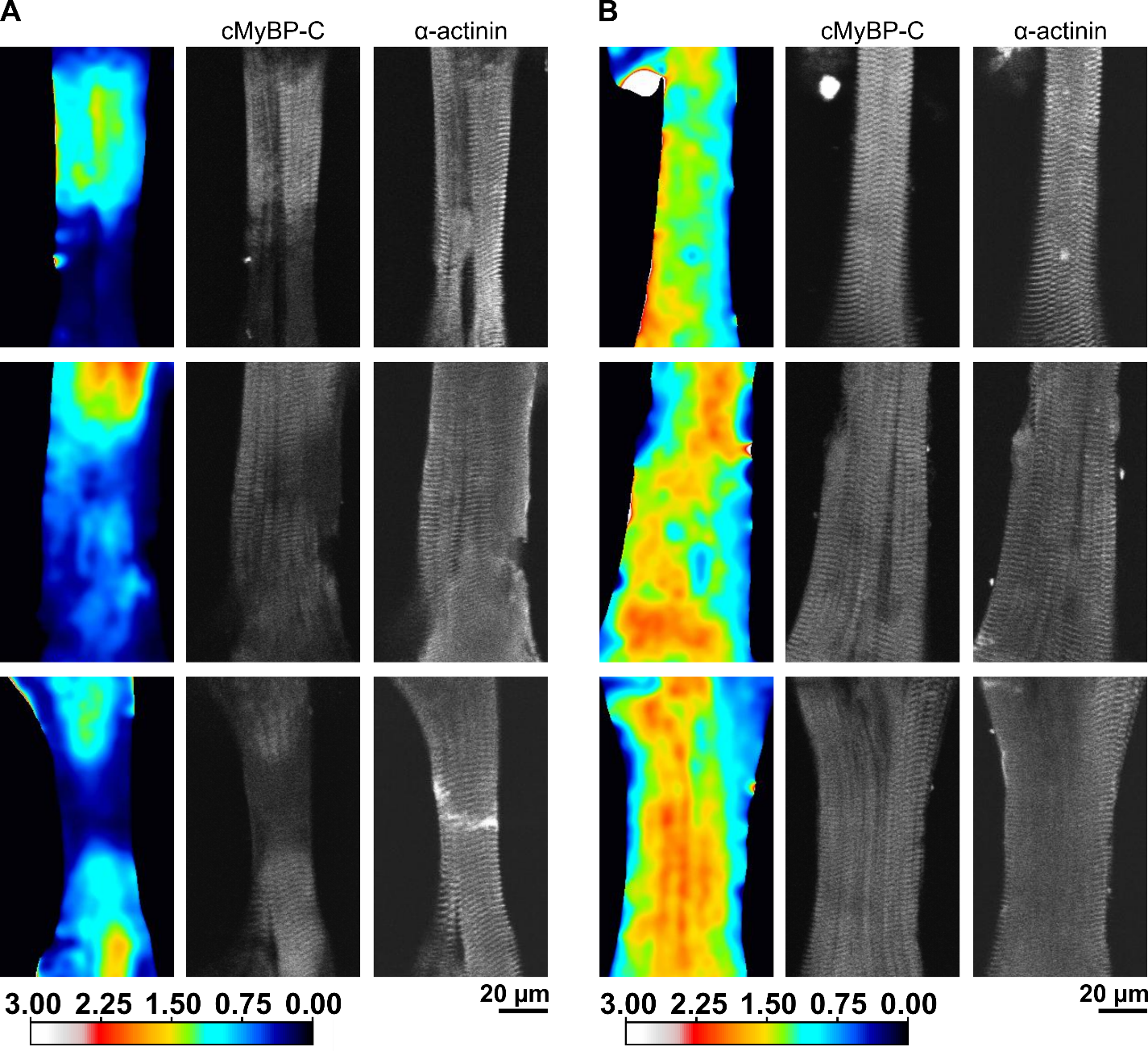
***

**Supplementary Figure 7**


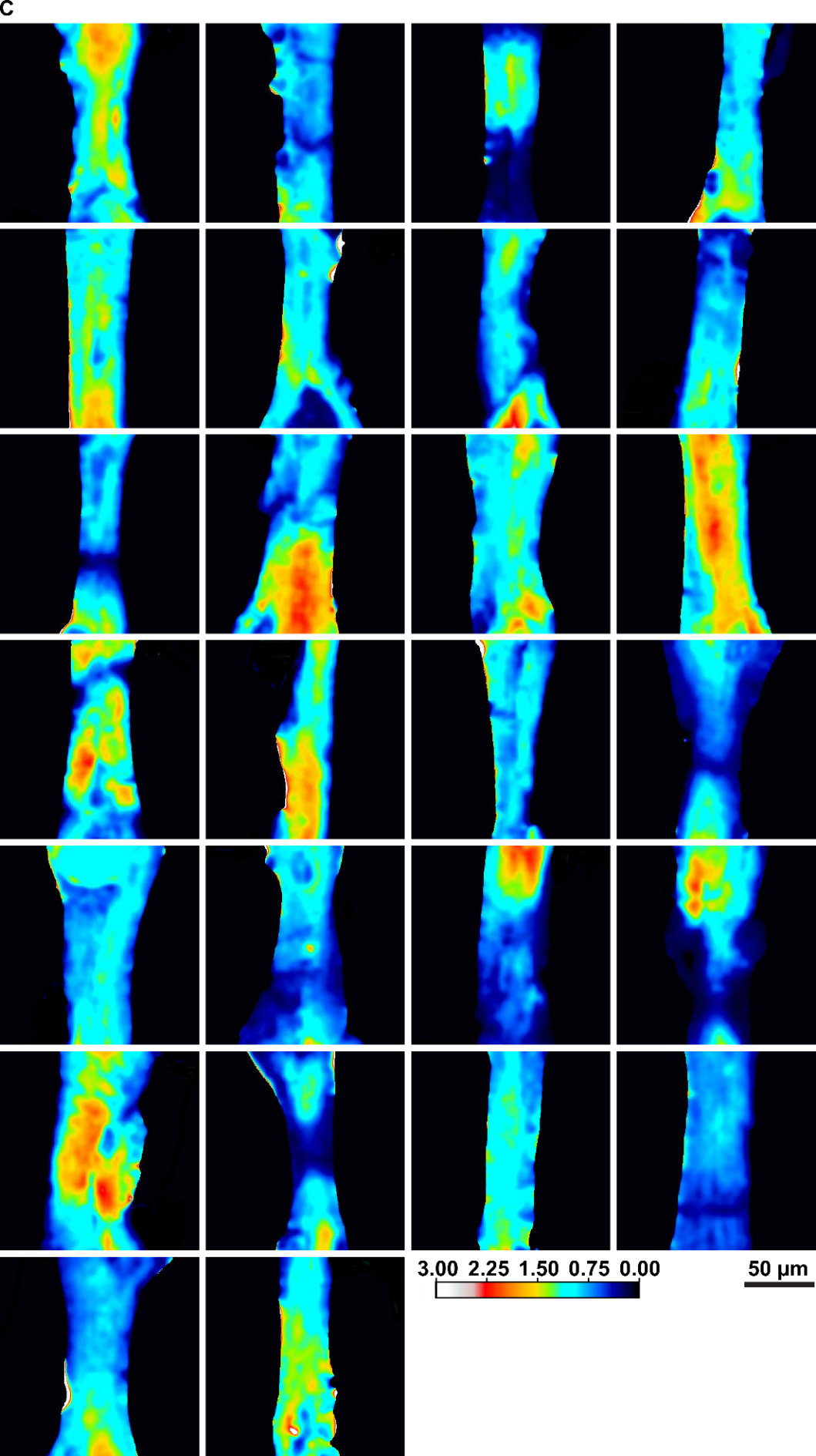


**Supplementary Figure 7**


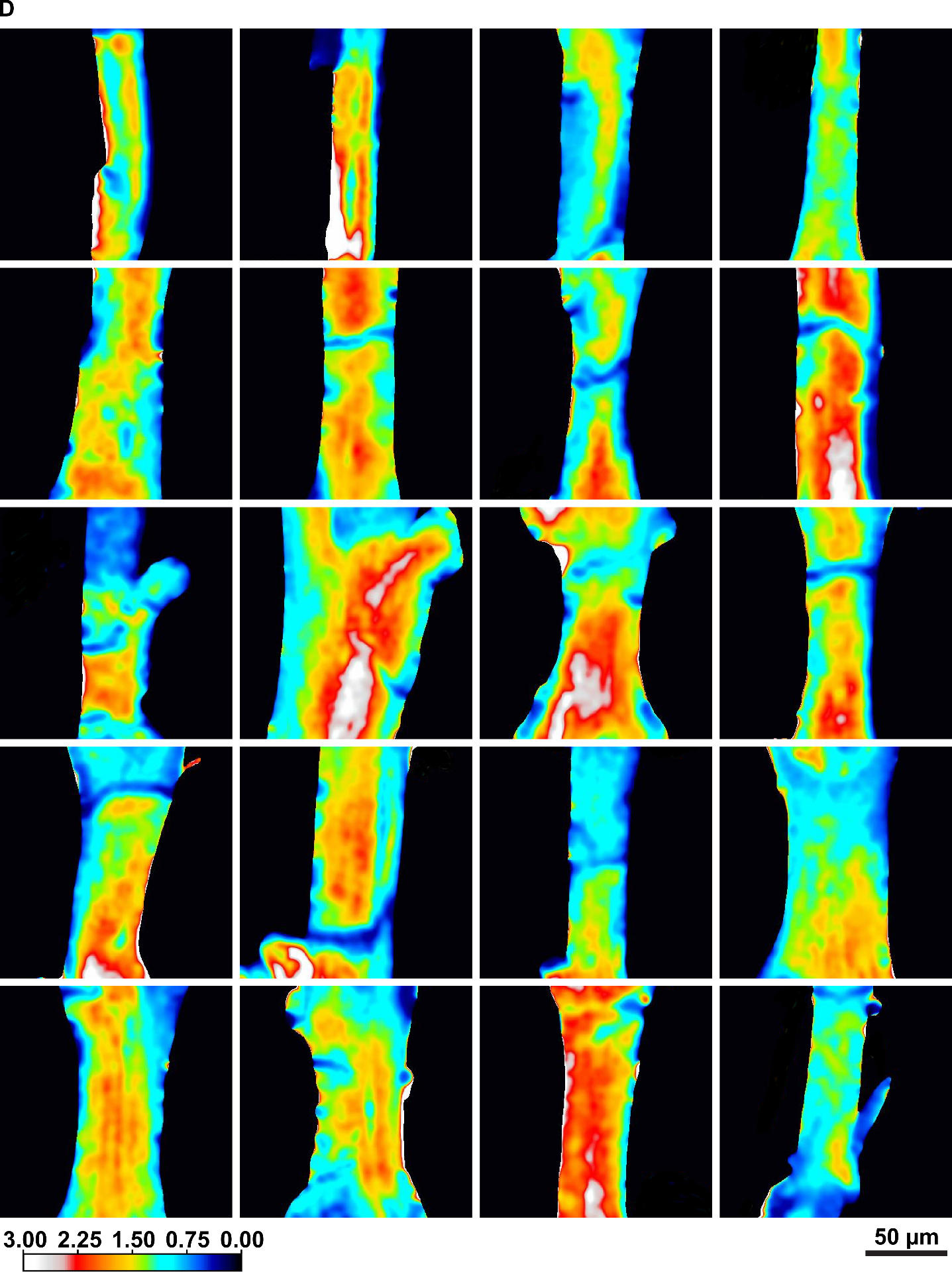


**Supplementary Figure 7: Pseudo-color display of the cMyBP-C to α-actinin intensity ratio in donor and cMyBP-C_trunc_ patient cardiomyocytes**

Individual cardiomyocytes from cMyBP-C_trunc_ patient and donor after force measurements were co-stained for cMyBP-C and α-actinin by specific antibodies. Images (longitudinal optical sections) of the cardiomyocytes mounted in the biomechanical setup were taken by confocal microscopy (Bio-Rad MRC600) of the center of the cardiomyocytes. **(A)** Three exemplary cMyBP-C_trunc_ cardiomyocytes and **(B)** donor cardiomyocytes each shown as grayscale image of cMyBP-C fluorescence (middle panels) and α-actinin fluorescence (right panels). The intensity quotient of cMyBP-C fluorescence to α-actinin (I_cMyBP-C_/I_α-actinin_) was calculated across the longitudinal optical section of the cardiomyocytes and is depicted in pseudo-color representation (left panels, dark blue, low I_cMyBP-C_/I_α-actinin_; red to white, high I_cMyBP-C_/I_α-actinin_). Overview of all cardiomyocytes analyzed in force measurements in pseudo-color representation for **(C)** cMyBP-C_trunc_ cardiomyocytes and **(D)** donor cardiomyocytes. Scale bar 20 µm in (A) and (B) and 50 µm in (C) and (D).


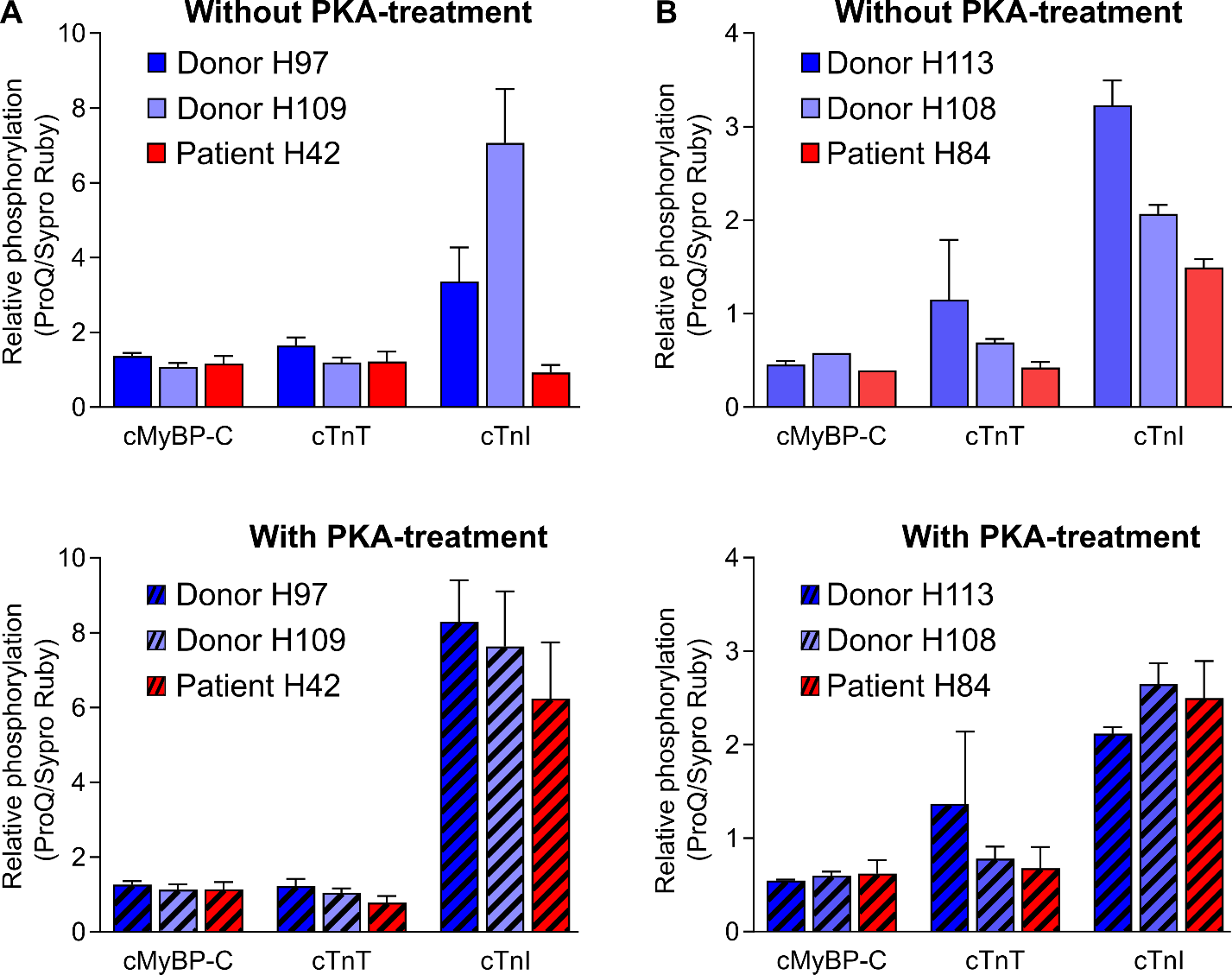


**Supplementary Figure 8: Phosphorylation levels in donor and HCM-patient cardiomyocytes before and after PKA-treatment**

Protein extracts from cardiac tissue of donors and HCM-patients were divided in two aliquots and left untreated (upper panel) or were treated with PKA and PP1 (lower panel). Proteins were separated by SDS-PAGE in two parallel gels for each approach. Phosphorylated proteins were stained by ProQ and subsequently total protein was stained by SyproRuby Diamond stain. Signal intensity was determined densitometrically and relative phosphorylation was calculated as ProQ/SyproRuby stain per protein. Treatment with PKA and PP1 equalized the ratio of phosphorylated proteins in all three samples. This becomes most evident for cTnI, where initial phosphorylation levels were highly divergent between the samples without treatment but increased to comparable levels upon PKA and PP1 treatment. **(A)** Analysis of two donors and a patient with a mutation in β-MyHC. **(B)** Analysis of two donors and a patient with a truncation mutation in cMyBP-C.


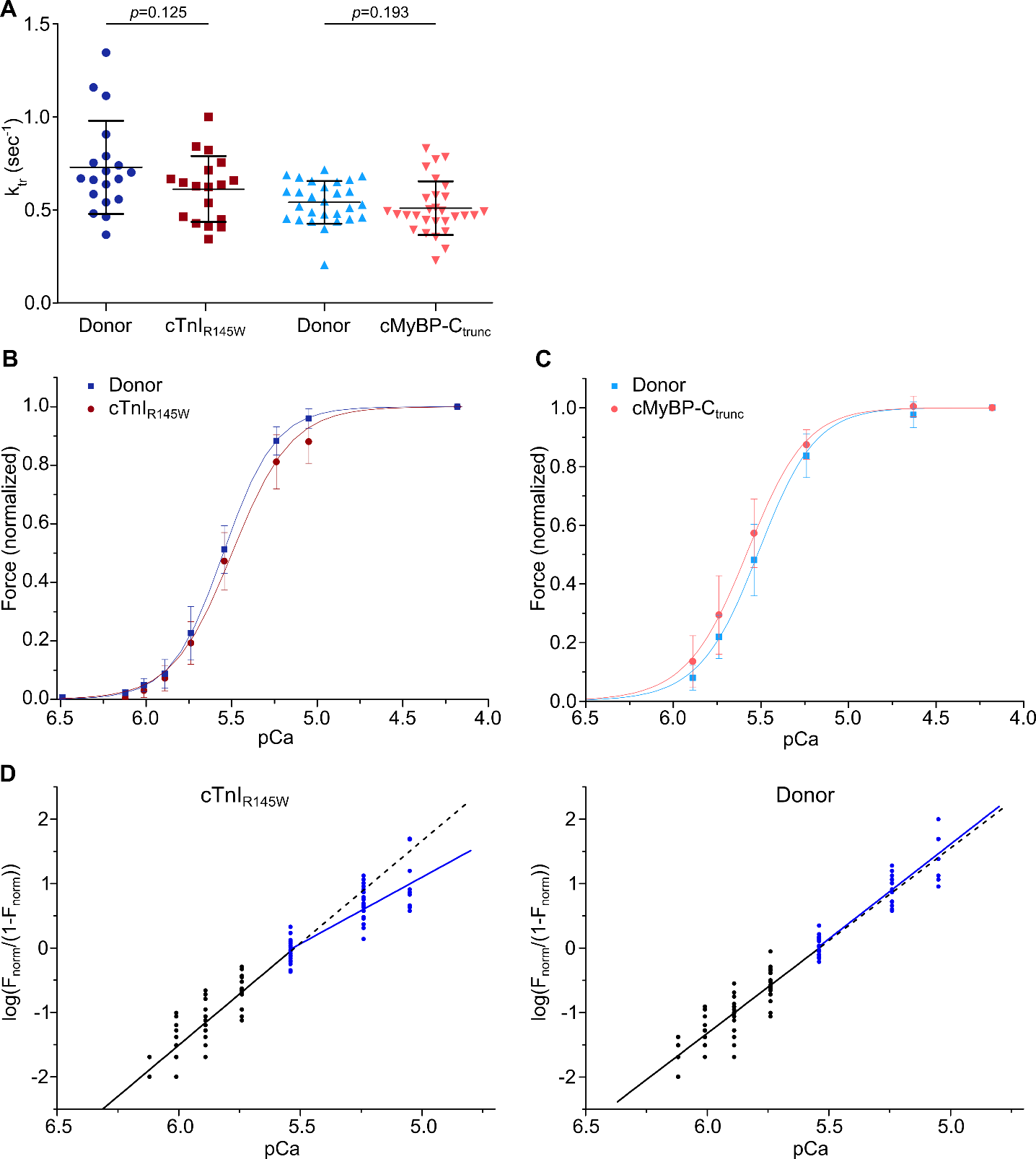


**Supplementary Figure 9: Force pCa relationships and cross-bridge cycling kinetics of cTnI_R145W_ and cMyBP-C_trunc_ cardiomyocytes**

Calcium dependent force generation of isolated, permeabilized single cardiomyocytes was determined for donors and HCM-patients with mutations in cTnI and cMyBP-C. **(A)** The rate constant of force redevelopment (k_tr_), reflecting cross-bridge cycle kinetics, was measured during isometric contraction at maximum activation after a quick release-restretch maneuver for single cardiomyocytes from donor (blue dots), cTnI_R145W_ and cMyBP-C_trunc_ patient tissue (red dots). *p*-values from *U* test are indicated in the figure. **(B)** and **(C)** mean force-pCa values from all measured single cardiomyocytes of donor (blue dots and lines) and patients (red dots and lines). cTnI_R145W_ cardiomyocytes shifted to the right; cMyBP-C_trunc_ cardiomyocytes shifted to the left. (B) cTnI_R145W_ cardiomyocytes (cTnI_R145W_ n = 20, donor n = 20) and (C) cMyBP‑C_trunc_ cardiomyocytes (cMyBP-C_trunc_ n = 29, donor n = 28); mean ± SD. **(D)** Force at different calcium concentrations from individual cTnI_R145W_ cardiomyocytes and donor cardiomyocytes were logit transformed and plotted against the respective pCa values. Values were fitted with two linear functions for values ≥ pCa 5.54 and ≤ pCa 5.54. Values close to 0 or 1 are highly affected by the transformation, therefore these values were left out of the fitting. pCa values between 6.12 and 5.54 can be described by linear regression (black line) for both, cTnI patient (R^2^= 0.86) and donor cardiomyocytes (R^2^= 0.88). For donor cardiomyocytes the fit at higher calcium concentrations (pCa 5.54 – 5.05; blue line, R^2^= 0.86) has an almost identical course as the extrapolation of the first linear fit. The slope of the fit at higher calcium of cTnI_R145W_ cardiomyocytes (blue line, R^2^= 0.7) differed significantly from the extrapolated linear force pCa relation.


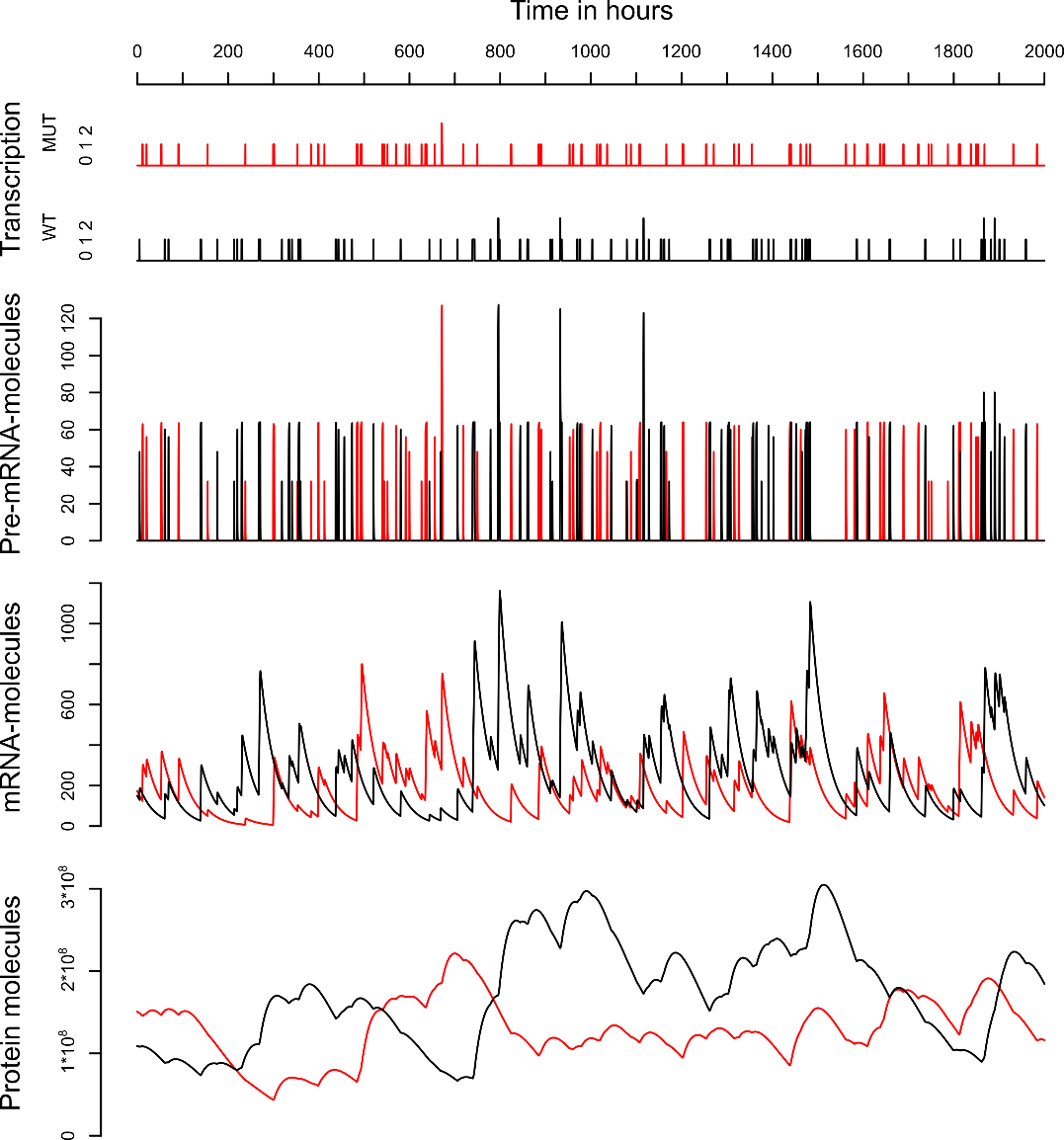


**Supplementary Figure 10: Simulated time-period of TNNI3 expression in a tetraploid cell from an HCM-patient with heterozygous TNNI3_c.433C>T_  mutation**

The predicted time course during the *TNNI3* expression in a tetraploid cell from an HCM-patient with the heterozygous *TNNI3*_c.433C>T_  mutation was simulated using the mathematical model. From top to bottom, panels show red lines for mutant allele and black lines for wildtype allele transcriptional bursts, pre-mRNA-molecules, mRNA-molecules and protein. Random on and off switching of allele transcription is indicated as the number of active alleles 0, 1 or 2 since a tetraploid cell contains two mutant and two wildtype alleles. The number of pre-mRNA, mRNA and protein molecules is calculated using literature data on *TNNI3*-mRNA and cTnI protein synthesis and degradation rates as stated in the Supplementary methods.


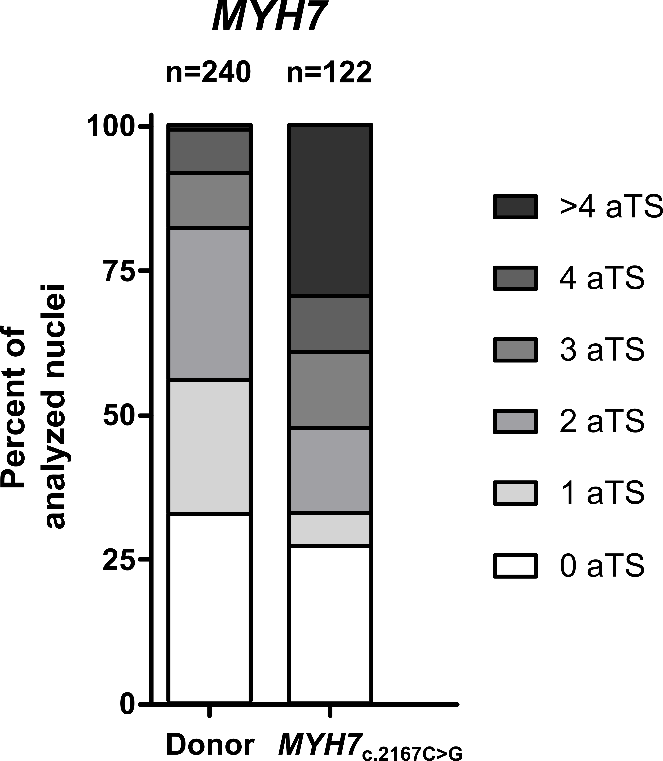


**Supplementary Figure 11: Quantification of aTS per cardiomyocyte nucleus for MYH7 in donor and HCM-patient cardiac tissue with mutation R723G (c.2167C>G)**

16 µm thick cryosections of *MYH7*_c.2167C>G_ patient tissue were hybridized with probe sets for intronic and exonic *MYH7*-RNA (1). The number of nuclei with 0, 1, 2, 3, 4 or >4 aTS per nucleus were divided by the total number of analyzed nuclei per individual, resulting in the displayed percentages of the six different groups for the biological samples. Both individuals show similar number of nuclei without aTS (33% for donor and 27% for HCM-patient). However, the HCM-patient also had nuclei with a higher number of aTS than the donor. The patient had higher fraction of nuclei with more than one aTS (about 70%) than the donor (about 45%).


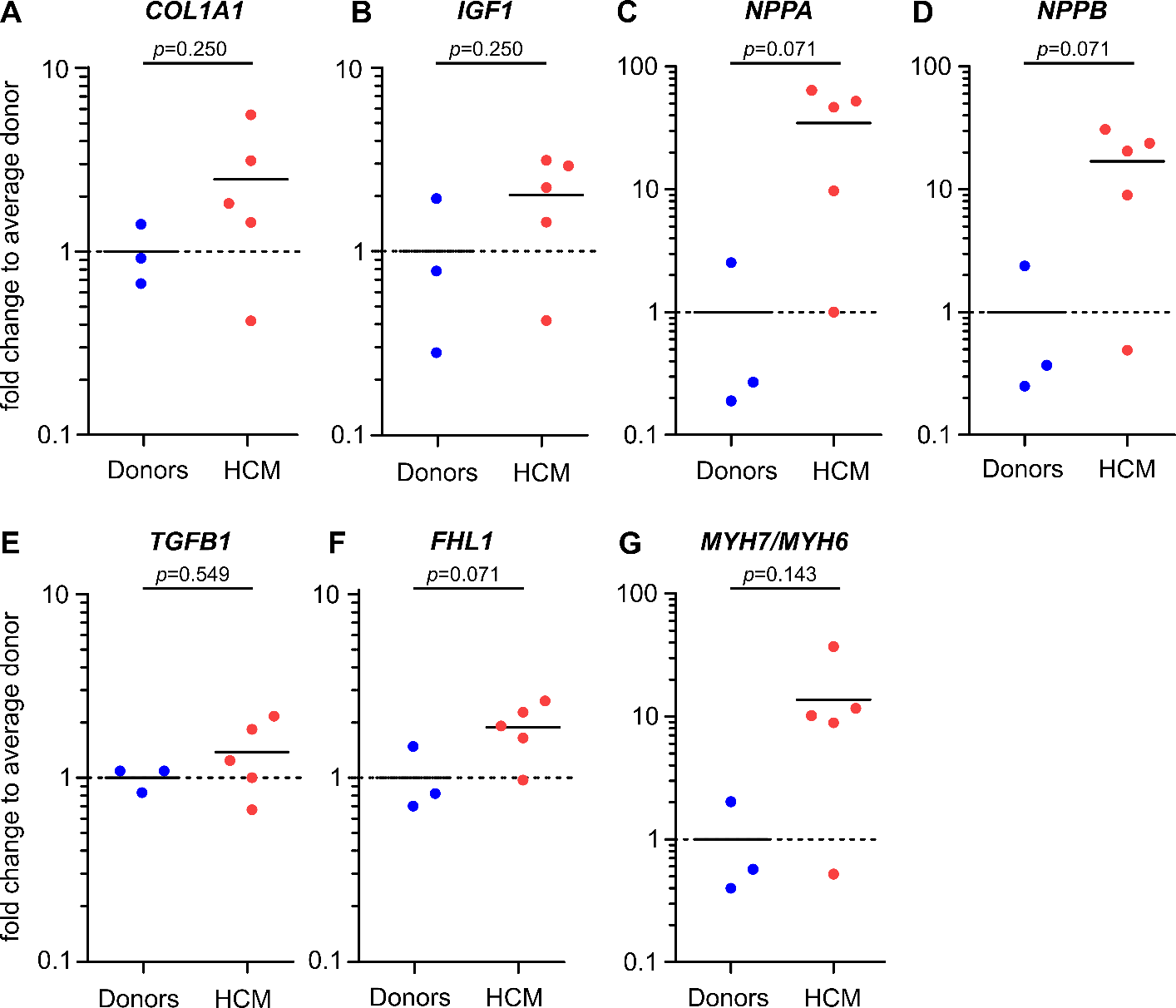


**Supplementary Figure 12: Upregulation of fibrosis and hypertrophy markers in HCM-patients compared to donors**

Total RNA was extracted from HCM-patient (N=5) and donor (N=3) tissue and reverse transcribed. We analyzed expression levels of marker genes for fibrosis (*TGFB1, COL1A1*) and hypertrophy (*IGF1, NPPA, NPPB,* and *FHL1*) in five HCM-patients relative to three donors by real-time PCR. Expression of *GAPDH, PGK1, POLR2A* and *RPL32* were used as reference for normalization. We found at least two-fold increased mean expression levels for *COL1A1* (2.5fold)*, IGF1* (2.1fold)*, NPPA* (35.8fold), *and NPPB* (16.9fold) in HCM-patients compared to donor samples **(A-D)**. Due to large inter-patient variability the measured upregulation was not statistically significant. *TGFB1* (1.4fold) and *FHL1* (1.9fold) also showed increased expression in HCM-patients, however below two fold increase **(E and F)**. In addition, *MYH6*-expression was downregulated leading to a shift in the *MYH7*/*MYH6* ratio towards *MYH7* (13.7fold increase) **(G)**. Each data point represents the mean of three independent RNA-extractions and subsequent quantifications. *p*-values of the *U* test are indicated in the figure.

**Description of the supplementary video material for cMyBP-C distribution**

Cryosections (5 or 10 µm) from cMyBP‑C_trunc_ patients and donor myocardium were stained with an N-terminus-specific antibody for cMyBP‑C (green) to visualize cell-to-cell cMyBP-C distribution. Co-staining with an α-actinin antibody (red) reveals sarcomeric Z-lines. Also, N-cadherin (white) and DAPI (blue) were stained. Z-stack images were taken by confocal laser scanning microscopy. 18 to 39 0.688 µm thick slices were imaged and imported into Imaris Viewer for 3D animation of the whole stack. Videos were recorded for two patients (c.2864_2865delCT, H36 and c.1458-6G>A, H45) and one donor (H89). The videos contain mixed views with different rotations. First of all channels merged, second only cMyBP-C and DAPI stain and third only α-actinin and DAPI stain. Cardiomyocyte striation is visible with both, cMyBP-C and α-actinin staining, however cMyBP-C staining shows cells and patches without signals, while α-actinin staining is more even.

**Supplementary methods:**

***Fluorescence in situ hybridization (FISH) for visualization of active transcription sites (aTS) and cytoplasmic mRNA in human heart tissue sections***

Active transcription sites were visualized as described previously (1). Briefly, two probe sets were designed using the Stellaris^®^ Probe Designer (https://www.biosearchtech.com/support/tools/design-software/stellaris-probe-designer), to hybridize with intronic and exonic sequence of *TNNI3* and *MYBPC3* (listed in Supplementary Table II; Stellaris^®^-probes; LGC Biosearch Technologies, Petaluma, CA, USA). Exonic sets were labelled with fluorophore Quasar 570 (LGC Biosearch Technologies) and intronic sets were labelled with fluorophore Quasar 670 (LGC Biosearch Technologies). For *TNNI3*, we designed 44 20-mers hybridizing to intronic sequences and 25 20-mers hybridizing to exonic sequences. For *MYBPC3* both, exonic and intronic probe sets contained the standard count of 48 20-mers.

FISH followed the protocol of Lyubimova et al. 2013 with some modifications (2). Frozen tissue sections (10-16 µm) were attached to HistoBond adhesive microscope slides (Marienfeld GmbH & Co. KG, Lauda-Königshofen, Germany) and fixed for 20-30 min with 4% PFA (in PBS) at room temperature. Tissue sections were washed three times with 1x PBS (w/o Mg^2+^, Ca^2+^). Sections were permeabilized for at least 1 h in 70% EtOH at 4°C and washed in wash buffer (10% formamide, 2x saline-sodium citrate (SSC) in nuclease-free water) for 2-5 min. Probes were applied in 100 µl of hybridization buffer (see (2) for detailed buffer information; 125 nmol/L of both probe sets per gene) and hybridized over-night at 37°C. Sections were washed twice for 30 min with wash buffer at 37°C. In the second wash step, DAPI was added at a concentration of 80 ng/mL. Sections were incubated in 2xSSC for 2-5 min at room temperature to remove excessive DAPI solution. 15 µL GLOX anti-fade buffer(2) were added and samples were stored on ice until imaging.

Sections were imaged with an Olympus IX83 fluorescence microscope with a 60x oil objective (ApoN TIRFMN.A. 1.49, Olympus, Tokyo, Japan) and a metal halide light source. Images were recorded with a cooled CCD camera (Orca-R^2^, Hamamatsu, Photonics, Japan). To analyze nuclei in total, three-dimensional z-stacks were recorded with motorized shutter and z-stage using filter sets for DAPI (Chroma U-F4900, Chroma Technology Corp, Bellows Falls VT, USA), GFP (Chroma U-F49002), Cy3 (Chroma U-F49004) and Cy5 (Chroma U-F49006). Exposure times for DAPI were 20 ms, 250 ms for GFP, 800 ms for Quasar 570, and 1 s for Quasar 670. Adjacent images in z-stacks were separated by 0.3 µm.

CellSens Dimension (version 1.16, Olympus, Tokyo, Japan) was used to count the number of active transcription sites (spots with co-localized fluorescence signal for intronic and exonic probe set in cardiomyocyte-nuclei). Striation patterns (bright field image) and/or cytoplasmic mRNA verified cells as cardiomyocytes.

***Control for specificity and sensitivity of RNA-FISH***

To control specificity of *MYBPC3* and *TNNI3* probe sets, tissue sections were treated with RNase A (10 µg/mL, Thermo Fisher Scientific, Dreieich, Germany) to remove all RNAs from the section (3). Tissue samples were fixed and permeabilized as described, incubated with RNase A for 30 min at 37°C, washed once with 1xPBS and then proceeded with FISH protocol as described above. In addition, sections from *Musculus soleus* from human control individuals were used which do not express *MYBPC3* or *TNNI3*. Hybridization and detection were performed as described above.

For the assessment of sensitivity, we used human pluripotent stem cell-derived cardiomyocytes to obtain cells with high expression levels of both genes. We used cell line MHHi001-A (4). Cells were differentiated into cardiomyocytes using published protocols (5). After differentiation in cardiac bodies for 15 days, the cells were dissociated using the StemDiff Kit (Stemcell) according to manufacturer’s instructions. Cells were cultivated on laminin (40 µg/ml; Life Technologies, Darmstadt, Germany) coated glass coverslips in bSF (basic serum free) medium, supplemented with 10% FCS (fetal calf serum, GE Healthcare Life Science, Freiburg, Germany) and 10 µM ROCK (Rho-associated protein kinase) inhibitor Y-27632 (Tocris). Medium was exchanged twice a week and cells were maintained in medium without ROCK-inhibitor.

For analysis of *MYBPC3*-transcription, cells were fixed in 4% PFA after eight days and subjected to RNA-FISH analysis. For analysis of *TNNI3*-transcription cells were incubated for eight days in standard medium and subsequently for seven days with 200 ng/ml triiodothyronine (T_3_). Medium was exchanged every day. Control cells were cultivated in parallel without T_3_. T_3_-treated cells and control cells were fixed in 4% PFA after treatment and subjected to RNA-FISH analysis.

***Single cardiomyocyte isolation by laser microdissection***

For the isolation of individual cardiomyocytes from flash frozen left ventricular samples, 5 µm cryosections were cut from the tissue using a Leica CM 1860 UV cryomicrotome (Leica Biosystems, Wetzlar, Germany). Sections were mounted on metal-frame slides covered with a 4 µm PEN-membrane (Leica) and were then fixed in ice-cold 75% ethanol and washed for 10 s in ice-cold PBS. Intercalated discs were visualized by staining for N‑cadherin. Tissue sections were incubated for 3 min at room temperature with 15 μl of the primary anti-N cadherin antibody (Sigma-Aldrich, C3678) diluted 1:10 in PBS. The slides were washed for 10 s in ice-cold PBS. The secondary anti-rabbit-TRITC antibody (Sigma-Aldrich), also diluted 1:10 in PBS, was applied for 3 min at room temperature. Cryosections were washed with ice-cold PBS for 10 s and then dehydrated in ice-cold ethanol (75% for 15 s, 95% for 30 s and 100% for 1 min). Isolation of single cardiomyocytes was performed by laser microdissection with a Leica LMD6 setup (Leica Microsystems, Wetzlar, Germany). Cardiomyocyte borders were identified by fluorescently labeled N-cadherin in the intercalated discs together with the striation pattern in the bright field image. Single cardiomyocytes were marked, laser dissected, and captured individually in the lid of a PCR-tube.

***Absolute quantification of TNNI3- or MYBPC3-mRNA copies in individual cardiomyocytes***

*TNNI3* or *MYBPC3*-mRNA per single cardiomyocyte was quantified by real-time PCR and copy numbers were calculated according to serial dilutions of standard-RNA. Standard-RNA was generated by *in vitro* transcription using *TNNI3-* or *MYBPC3*-cDNA as template for the T7-MegaScript Kit (Thermo Fisher Scientific) according to the suppliers’ manual. The *TNNI3*-template DNA was produced by PCR with plasmid containing *TNNI3*-cDNA (Sequence is given in Supplementary Table IV) with a forward primer equipped with the T7 promotor sequence and a corresponding reverse primer (*TNNI3 in vitro* transcription forward and reverse, Supplementary Table III). PCR mix contained 1x reaction buffer, 0.2 mmol/L of each dNTP (Fermentas), 0.2 μmol/L of both primers *TNNI3 in vitro* transcription forward and reverse (Supplementary Table III), and 0.04 U/μl HotStarTaq (Qiagen) in a final volume of 50 μl. After initial activation for 15 min at 95°C, 40 cycles were applied with 95°C for 30 s, 54.7°C for 30 s, and 72°C for 30 s and final elongation at 72°C for 2 min. *MYBPC3*-cDNA was ordered as gBlock from IDT (Integrated DNA Technologies, Coralville, IA, USA) already equipped with T7 promotor sequence (Sequence is given in Supplementary Table IV). *In vitro* transcribed RNA was isolated using the PeqGold Total RNA Kit (VWR) according to the suppliers’ instructions. RNA concentration was determined photometrically using a NanoDrop (PeqLab, Erlangen, Germany) and used to calculate copy numbers per µl.

Single donor cardiomyocytes were isolated by laser microdissection and after cell lysis, mRNA was reverse transcribed in parallel with serial dilutions of the standard-RNA. A final volume of 13.5 µl cDNA reaction mix (0.125 mmol/L dNTPs (Fermentas, Vilnius, Lithuania) each, 0.5 μmol/L *TNNI3* RT or *MYBPC3* RT (Supplementary Table III), 1x reaction buffer, 1 U/μl RNase inhibitor (RiboSafe, Bioline) and 5 U/μl reverse transcriptase (Tetro, Bioline)) was added to the cells or to 1 µl standard RNA, respectively, and incubated at 42°C for 1 h on a micro mixer (6). For pre-amplification, 40 µl of PCR reaction mix (1x reaction buffer, 0.2 mmol/L of each dNTP (Fermentas), 0.2 μmol/L of both primers *TNNI3* real-time F and *TNNI3* real-time R1 or *MYBPC3* real-time F1 and *MYBPC3* real-time R1 (Supplementary Table III), 4% DSMO and 0.04 U/μl HotStarTaq (Qiagen)) were added to the cDNA. Initial activation was performed for 15 min at 95°C followed by 10 cycles with 95°C for 30 s, 59.7°C for 30 s, and 72°C for 30 s. Final elongation was performed at 72°C for 2 min. For real-time PCR, 1 µl PCR-product was added to 1x PowerUp SYBR Green Master Mix (Applied Biosystems, Foster City, CA, USA) and 0.25 µmol/L *TNNI3* real-time F and *TNNI3* real-time R2 (Supplementary Table III) in a final volume of 20 µl in duplicates. For *MYBPC3* quantification, we used a FAM labeled, NFQ-MGB quenched qPCR probe (0.0625 µmol/L*, MYBPC3* probe in Supplementary Table III) in 1x TaqMan Universal Master Mix II, no UNG (Thermo Fisher Scientific) with 0.25 µmol/L *MYBPC3* real-time F2 and *MYBPC3* real-time R2 (Supplementary Table III) in a final volume of 20 µl in duplicates. PCR-protocol was identical for both genes. Initial denaturation was performed at 95°C for 10 min followed by 40 cycles of 95°C for 15 s and 60°C for 45 s using QuantStudio™ 6 Flex System (Thermo Fisher Scientific).

Linear regression analysis of the standard-RNA was used to calculate the copy number per cardiomyocyte. Serial dilution of *in vitro* transcribed *TNNI3-*mRNA was done in steps of 2*10^6^, 2*10^5^, 2*10^4^, 2*10^3^, 2*10^2^, and 2*10^1^ copies. For *MYBPC3*-mRNA quantification, we used a serial dilution with 2*10^6^, 2*10^5^, 2*10^4^, 1*10^4^, 2*10^3^, 1*10^3^, and 2*10^2^ copies. We experienced difficulties to determine signals in reactions with copy numbers below 2*10^2^ copies. However, measured cardiomyocytes presented robust signals below this point of the standard curve. Extrapolation of copy number per cell from the standard curve revealed no signals, which would have represented copy numbers below zero copies per cell. This indicates experimental limitations of synthetic *MYBPC3*-mRNA dilution probably due to adherence at plastic reaction tubes, which is not present in whole cell lysates. We therefore extrapolated low copy numbers of each single cardiomyocyte from the standard curve. As single cardiomyocytes were isolated from 5 µm cryosections, these isolates represent only parts of the whole cell. Taking into account an average cardiomyocyte diameter of 17 μm(7), the mRNA-copy number per cell was calculated as three-fold of the originally determined copies. If the number of molecules per cell is not following a Poisson distribution, indicated by equal values for mean and variance, this suggests burst-like transcription as previously observed (8).

***Relative quantification of mutant to WT TNNI3-mRNA by RT-PCR***

A total volume of 20.5 μl annealing reaction mix (0.125 mmol/L dNTPs (Fermentas) each, 0.5 μmol/L *TNNI3* RT (Supplementary Table II)) was added to each individual laser-microdissected cardiomyocyte in the lid of a tube. Samples were incubated at 65°C for 5 min. Reverse transcription mix was completed by adding 6.5 µl containing 1x reaction buffer, 1 U/μl RNase inhibitor (RiboSafe, Bioline, London, UK) and 5 U/μl reverse transcriptase (Tetro, Bioline). For subsequent cDNA-synthesis, samples were incubated on a micro mixer(6) for 1 h at 42°C. For nested-PCR, cDNA samples were split in two aliquots of 13.5 μl each and 40 µl PCR reaction mix (1x reaction buffer, 0.2 mmol/L of each dNTP (Fermentas), 0.2 μmol/L of both primers *TNNI3* F1 and R2 (Supplementary Table II), 4% DMSO and 0.04 U/μl HotStarTaq (Qiagen, Hilden, Germany) in a final volume of 53.5 μl) was added. Amplification was performed by initial activation for 15 min at 95°C followed by 30 cycles with 95°C for 30 s, 59.7°C for 30 s, and 72°C for 30 s. Final elongation was carried out at 72°C for 2 min. For the second nested PCR, 1 μl of the PCR-product was used as template. PCR reaction mix contained 1x reaction buffer, 0.2 mmol/L of each dNTP (Fermentas), 0.2 μmol/L of both *TNNI3* F2 and R2 (Supplementary Table II), 4% DMSO, and 0.04 U/μl HotStarTaq (Qiagen) in a final volume of 25 μl. Following an initial activation for 15 min at 95°C, 35 cycles were applied with 95°C for 30 s, 56.2°C for 30 s and 72°C for 30 s. The final elongation was performed at 72°C for 2 min. Possible heteroduplex-formation was reduced by a reconditioning PCR(9) using 2.5 μl of the second nested PCR-product in a final volume of 25 μl PCR reaction mix identical to the second nested PCR, and the same PCR-protocol was run for four cycles. Reconditioned PCR-products were treated with *Mwo*I for mutation R145W or *Bbs*I for the single nucleotide polymorphism (SNP), respectively. 0.12 µl *Mwo*I or 1.2 µl *Bbs*I restriction enzyme (both New England BioLabs, Ipswich, MA, USA) and 1.2 µl of the respective restriction buffer were added to 12 μl PCR-product and incubated at 60°C for 15 min (*Mwo*I) or 37°C for 3 h (*Bbs*I). Heat inactivation of *Bbs*I was performed at 65°C for 20 min. For mutation R145W, treatment resulted in a 115 base pair (bp) band for both alleles, a 202 bp fragment for the mutant and 160 bp and 42 bp fragments for the wildtype (WT) allele, respectively. For the SNP (rs3729841), treatment yielded two fragments 273 and 44 bp long for the SNP-allele and left the WT-allele uncut as a 317 bp fragment. *Mwo*I or *Bbs*I-treated PCR-products were separated on 3% sieving agarose (Biozym) gels stained with ethidium bromide. Quantification of transcripts from the *TNNI3*-alleles was performed as described previously in detail.^30^ Briefly, the IOD of the restriction fragments was determined densitometrically using the TotalLab (Newcastle upon Tyne, Great Britain) and Origin (OriginLab, Northampton, MA, USA) software. To correct for the intercalated stain by ethidium bromide, the IOD was normalized against the number of bases of the respective fragment. The fraction of mutant *TNNI3*-mRNA was calculated as the ratio of IOD/bp of the mutation-specific fragment over IOD/bp of the fragment generated by both mutant and WT *TNNI3*-mRNA. In case of the SNP-analysis, no common fragment is available after restriction digestion for normalization. The fraction of SNP-allele was calculated from the ratio of IOD/bp of the SNP-specific fragment over the sum of both SNP and WT *TNNI3*-mRNA IOD/bp ratios.

***Control of linearity***

We tested the linearity of the relative quantification of *TNNI3* as previously described^30^ using standard plasmids that encoded for the WT *TNNI3* and the R145W cDNA sequence. Defined mixtures of different ratios WT/R145W (10/90; 30/70; 50/50; 70/30; 90/10) were diluted to a concentration of 1*10^3^ copies/µl and used as templates for nested PCR, reconditioning PCR and subsequent allele-specific restriction analysis. Densitometric quantification of the restriction products on agarose gels was performed as described for the single cell analysis above.

***Quantification of experimental scatter by multialiquot control***

We determined the experimental scatter of our single cell reverse transcription-PCR procedure with a multialiquot control as described previously (1). Total RNA was extracted from five donor cryosections with the PeqGold Total RNA Kit (VWR, Radnor, PA, USA) and diluted serially. After reverse transcription-PCR as described for the single cardiomyocytes, the signal intensity from the diluted RNA was compared to that single cardiomyocytes. A dilution of the total RNA with a similar IOD compared to that of single cardiomyocytes was chosen, which presented a variability of the signal intensity between individual aliquots comparable to that of the individual cardiomyocytes. The diluted total RNA was separated into 20 aliquots. RT-PCR, reconditioning PCR and allele-specific restriction analysis was performed in parallel using the same protocol as for single cardiomyocytes. The IOD of the SNP- and WT-fragments was determined and the ratio of SNP-allele was calculated as described for the single cardiomyocytes.

***Protein quantification via western blot***

Truncation mutations in cMyBP-C most often lead to nonsense-mediated decay of mutant mRNA and protein. To test whether the cMyBP-C_trunc_ patient expresses the truncated protein, we performed western blot analysis. Relative cMyBP-C protein amounts were quantified from donor and cMyBP-C_trunc_ patient interventricular septum (IVS) samples via SDS-PAGE and subsequent western blot analysis. Samples were ground in a cryo-mortar at -196°C. A final concentration of 80 µg/µl of the samples was achieved and prepared with RothLoad1 K929.1 (1 to 1 with H_2_O). 5 µl of the solution were loaded onto 10% SDS-polyacrylamide gels (separating gel: 10% acryl-/bisacrylamide 30:08 (Roth)), 0.375 mol/L tris-base (Sigma-Aldrich) pH 8.8, 0.2% SDS (Serva), 0.1% APS (Sigma-Aldrich), 0.05% TEMED (Roth); collecting gel: 3% acryl-/bisacrylamide 30:08, 0.125 mol/L tris-base pH 8.8, 0.2% SDS, 0.1% APS, 0.05% TEMED) together with a molecular weight marker (Precision Plus Protein™ Dual Color). Gel electrophoresis was performed in precooled (4°C) running buffer (0.19 mol/L glycine (Carl Roth, Karlsruhe, Germany), 25 mmol/L tris-base and 3.5 mmol/L SDS) at room temperature. 20 mA were applied for 20 min followed by 25 mA for 70 min. The gel was then blotted onto a nitrocellulose membrane (0.2 µm, GE Healthcare, Chalfont St Giles, Great Britain). A transfer buffer containing 0.19 mol/L glycine (Carl Roth), 25 mmol/L tris-base (Sigma-Aldrich) and 10% methanol (Roth) was used at 4°C at 30 V for 90 min. To visualize the total protein content of each lane, the membrane was stained with Ponceau (Merck) and staining was documented. Antibody staining was performed after washing the membrane with TBS-T for 5 min at room temperature. The membrane was blocked with 5% milk powder (sc2325, Santa Cruz, Dallas, TX, USA) in TBS-T (150 mmol/L NaCl (Merck) and 50 mmol/L tris-base, 0,1% Tween 20 (Sigma-Aldrich) pH 7,4 for 1 h and primary N-terminal cMyBP-C antibody (Santa Cruz, sc137237, diluted 1:500 in 5% milk in TBS-T) or α-actinin (Abcam, ab9465, diluted 1:1000 in 5% milk in TBS-T) was applied over night at 4°C. After washing three times for 5 min with TBS-T again, secondary antibody goat anti mouse (BioRad 172-1011, diluted 1:3000 in 5% milk in TBS-T) or donkey anti rabbit (GE Healthcare, NA934V, diluted 1:3000 in 5% milk in TBS-T) was applied for 1 h at room temperature. The membrane was washed three times for 5 min with TBS-T and once with TBS and then secondary antibody was visualized with LAS4000 system (ImageQuant, GE Healthcare). The integrated optical density of cMyBP‑C and α‑actinin bands from antibody staining was determined with ImageQuant 1D (GE Healthcare).

***Protein staining of cryosections***

For an overview of cMyBP-C protein distribution within the tissue of patients and donors, 5 or 10 µm cryosections from cMyBP-C_trunc_ patient left ventricular tissue and donor tissue were co-immunostained for cMyBP-C, α-actinin and N-cadherin or cMyBP-C and β-MyHC. In detail, cryosections were obtained using HistoBond adhesive microscope slides (Marienfeld GmbH & Co.KG) and immediately fixed in 4% paraformaldehyde for 1 h at room temperature. Cryosections were washed with PBS, permeabilized with 0.5% Triton X-100 in PBS for 30 min and washed again with PBS. A blocking solution of 5% bovine serum albumin (BSA; Gerbu Biotechnik GmbH) in PBS was applied for 30 min at room temperature and then primary antibodies were incubated simultaneously. We used anti-cMyBP‑C (Abcam, ab262964, from rabbit, dilution 1:800 or 1:400 in 5% BSA), anti-α-actinin (Abcam, ab9465, from mouse, dilution 1:100 in 5% BSA), anti-β-MyHC (Sigma Aldrich, M8421, from mouse, dilution 1:100 in 5% BSA) and anti-N-cadherin (antibodies-online GmbH, ABIN1440032, from goat, dilution 1:100 in 5% BSA) antibodies. For epifluorescent microscopy, cryosections were incubated for 1 h and for confocal microscopy over night at room temperature. The specificity of anti-cMyBP-C antibody was confirmed by stainings of *M. soleus* (cardiac specificity), as well as line profiles throughout the sarcomeric pattern and additional co-stainings against β-MyHC. After washing three times for 5 min with PBS, secondary antibodies Alexa Fluor 488 (Life Technologies, A21206, donkey anti rabbit, dilution 1:400 in 5% BSA), Alexa Fluor 555 (Life Technologies, A31570, donkey anti mouse, dilution 1:400 in 5% BSA) and Alexa Fluor 680 (Life Technologies, A32860, donkey anti goat, dilution 1:400 in 5% BSA) were incubated for another hour at room temperature. Secondary antibodies were removed by washing with PBS for three times for 5 min. DAPI (Sigma-Aldrich, D9542, dilution 1:12500 in PBS) was used to stain nuclei. In the end, cryosections were covered with Fluoroshield (Sigma-Aldrich, F6182) and a small glass coverslip and analyzed by either epifluorescent microscopy at an Olympus IX83 inverted microscope or confocal laser scanning microscopy at a Zeiss LSM microscope.

***Immunofluorescence protein staining in individual cardiomyocytes***

After calcium dependent force measurements, cardiomyocytes from cMyBP-C_trunc_ patient and donor left ventricular tissue were co-immunostained for cMyBP-C and α‑actinin. The cMyBP-C protein was detected by an N-terminal anti-cMyBP-C antibody from mouse (sc137237; Santa Cruz) and α-actinin with an anti-α-actinin antibody from rabbit (ab137346; Abcam, Cambridge, United Kingdom). Both first antibodies were diluted 1:10 in relaxation solution for incubation of the mounted cardiomyocytes for 30 min. Cells were washed twice with relaxation solution for 5 min and incubated with the corresponding secondary antibody (TRITC – anti mouse for cMyBP-C; 1:10 dilution (T5393, Sigma-Aldrich) and Alexa Fluor 488 – anti rabbit for α-actinin; 1:20 dilution; (A11008, Life Technologies, Carlsbad, CA, USA)) in relaxation solution for 30 min. Cells were washed with relaxation solution twice for 5 min. Microscopy of the mounted cardiomyocytes was performed with a confocal microscope (Bio-Rad MRC600) that was part of the setup for the calcium dependent force measurements. One plane in the vertical center of the cardiomyocyte was imaged to obtain a longitudinal optical section of the middle of the cardiomyocyte. A precise protocol of the imaging procedure was applied for all cardiomyocytes to avoid bleaching based artifacts. First imaging occurred for cMyBP‑C with 568 nm wavelength and then for α-actinin with 488 nm wavelength at constant exposure times.

***Phosphorylation analysis***

Phosphorylation of sarcomeric proteins was analyzed by ProQ/SyproRuby staining as described previously (10). In brief, cardiac tissue was homogenized in liquid nitrogen and either stored or re-frozen immediately after PKA treatment. Frozen tissue was resuspended in 1 ml cold 10% trichloroacetic acid (TCA), 0.1% (w/v) dithiothreitol (DTT) in acetone to maintain protein phosphorylation and solubilized in sample buffer containing 15% glycerol, 62.5 mM Tris (pH 6.8), 1% (w/v) SDS and 2% (w/v) DTT. Proteins were separated on 4-15% gradient gels (Criterion, BioRad). Phosphorylated proteins were stained by ProQ Diamond stain and subsequently total protein was stained with SYPRO-Ruby (both Life Technologies, Darmstadt, Germany) according to the manufacturer’s protocol and integrated optical density (IOD) was determined using a LAS4000 system (ImageQuant, GE Healthcare). IODs of proteins of interest were normalized to IOD of α‑actinin to correct for loading differences and to a control sample to correct for inter-gel differences in staining intensity. Phosphorylation levels were calculated as ProQ/SyproRuby using corrected IODs for cMyBP-C, cTnT, and cTnI, respectively.

***Force measurements of single cardiomyocytes***

Single cardiomyocytes were isolated mechanically with a modified approach. The original method^15^ was optimized for small sample sizes, down to 1 mm³ of cardiac tissue. For that the tissue was quickly thawed in calcium free storage solution (5.95 mmol/L sodium ATP, 6.04 mmol/L MgCl_2_, 2.0 mmol/L EGTA. 139.6 mmol/L KCl, 10 mmol/L imidazole). Immediately after this step, the tissue was dispersed with a T8 ULTRA-TURRAX® drive (IKA®-Werke, Staufen, Germany) with a 3.8 mm rotor at 5000 min^-1^ three times for 1 s. The resulting cardiomyocyte cell suspension was stored at 4°C for up to four days and each day 1 mmol/L freshly prepared DTT was added. The air above the solution was replaced with nitrogen and tightly sealed to reduce oxidative processes. A protease inhibitor cocktail was added to the storage solution as described previously (10). Single, cylindrically shaped cardiomyocytes showing striation pattern were selected from the cardiomyocyte suspension, and attached to a cantilever (High Speed Length Controller, Aurora Scientific, Canada) and force transducer with silicone adhesive (Aqua Sil, Den Braven, Oosterhout, Netherlands). Mounted cardiomyocytes were incubated with 0.5% triton to remove residual cell membrane fragments and the sarcomere length was adjusted to 2.2 µm. Excess triton was washed off by incubation two or three times in fresh relaxing solution (pCa 9.0, see below) for 3 min. Phosphorylation levels of sarcomeric proteins were adjusted in patient and donor cardiomyocytes by incubation with protein phosphatase 1‑alpha (PP1, Sigma-Aldrich, St. Louis, MO, USA) for 60 min followed by incubation with protein kinase A (PKA, Sigma-Aldrich) for 40 min, both at 20°C in relaxing solution.

Cardiomyocyte force generation was measured in activating solutions of different calcium concentrations, depicted as pCa values (-log10[Ca^2+^]), as described previously (10). Several force levels between relaxing conditions (pCa 9.0: 3 mmol/L EGTA, 10 mmol/L imidazole, 2 mmol/L MgCl_2_, 10 mmol/L caffeine, 10 mmol/L CrP, 2 mmol/L MgATP and 110 mmol/L K-propionate) and saturating calcium concentrations (pCa 4.63, 3 mmol/L CaEGTA, 10 mmol/L imidazole, 2 mmol/L MgCl_2_, 10 mmol/L caffeine, 10 mmol/L CrP, 1 mmol/L MgATP, 110 mmol/L K-propionate) were measured. Activating solutions with different pCa values were obtained by mixing relaxing and activating solutions in different proportions, calculated using the program “calcium” (11). pH of all solutions was adjusted to 7.1 at 15°C. Chemicals were purchased from Sigma-Aldrich or Merck (Darmstadt, Germany).

The protocol for the force measurements was as previously published (1,10). The mounted cardiomyocyte was moved from relaxing solution to different activating solutions and force generation was recorded. At the highest calcium concentrations, seven quick release/restretch maneuvers were applied to stabilize the sarcomere structure of the cells until the force reached a steady state level (1). Each maneuver consisted of a quick release to 40% of initial length followed after 70 ms by a restretch to 10% overstretched length and back to the initial length. At submaximal calcium concentrations a slightly different protocol was used with one quick release/restretch maneuver after the cell reached the maximum isometric force at this pCa value (10). This protocol was repeated for the submaximal activating solutions containing different calcium concentrations in a random order, to determine force-pCa relations. Force run-down was determined at the end of all measurements by a second maximum activation, which was compared to the initial maximum activation. Cardiomyocytes with a force run-down >20% were excluded from the analysis. All force values were normalized for the cardiomyocyte cross-sectional area. The width and height of the analyzed cardiomyocyte was measured as described previously^17^ and the cross-sectional area was calculated assuming an elliptical shape of the cardiomyocyte cross section.

Total force (F_tot_) was determined as difference between isometric steady state force and zero force after quick release. Passive force (F_pass_) was determined after the cardiomyocyte was transferred to relaxing solution. After full relaxation of the cardiomyocyte, it was slackened for 10 s by a release of 30% of cell length (10). The difference of force between the state after release and the slackened state represents the passive force. Finally, the active force was calculated as F_act_= F_tot_-F_pass_.

***Fitting of force-pCa relationships***

The active forces of each isolated cardiomyocyte, generated at different calcium concentrations (pCa-values) were normalized to the maximum force at saturating calcium concentration (pCa 4.18). pCa50 (the pCa-value for 50% maximum force) and Hill coefficient (slope at pCa50) were determined by least-squares fitting of a Hill equation to the normalized force values at different pCa-values.

For determining differences in the increase of force with rising pCa, the normalized force values were logit transformed using the equation:

Logit(F) = log(F/(1-F)), where 0<F<1.

***Gene expression analysis by real-time PCR***

Cryopreserved left-ventricular heart tissue was cryo-sectioned at a thickness of 5 µm into a series of 10 to 30 cryosections for an effective sample mass of up to 10 mg. Total RNA was purified and eluted in 90 µl nuclease-free water using the Monarch® Total RNA Miniprep Kit (New England BioLabs), followed by DNaseI-treatment using 3 U DNaseI (New England BioLabs) in 100 µl total reaction volume at 37°C for 30 min. RNA was enriched using the Monarch® RNA Cleanup Kit (New England BioLabs) and elution in 9 µl nuclease-free water. 50 ng RNA were reverse transcribed in 10 µl total reaction volume containing 2.5 µM random decamers (Thermo Fisher Scientific), 5 U TETRO reverse transcriptase (Bioline), 1 U RNase inhibitor (Bioline) and 500 µM dNTPs (Fermentas) incubated for 1 h at 41°C.

Quantitative real-time PCR was performed in duplicates of 20 µl total reaction volume each containing 1 µl cDNA template (1:3 diluted) and 0.5 µM of each forward and reverse primer (Supplementary Table III) in 1x PowerUp™ SYBR® Green Master Mix (Thermo Fisher Scientific). Reactions were run on a QuantStudio™ 6 Flex System (Thermo Fisher Scientific) alongside a non-template control (NTC) with initial denaturation at 95°C for 10 min followed by 40 cycles of 95°C for 15 s and 60°C for 45 s. Primers were designed using Primer-BLAST(12) and experimentally validated for specificity and efficiency prior to the experiments. Likewise, effective DNaseI-treatment was tested using cDNA-synthesis without reverse transcriptase and a primer set for the amplification of genomic DNA (data not shown).

The entire procedure was repeated three times for each individual sample. Two of three RT reactions using template RNA from patient H51 were based on subsamples of the same RNA extraction due to limited tissue availability. Each dataset was quality-assessed by examination of NTC signals and melt curve plots. Fold change was calculated using the extended dCT method (13). In brief, the arithmetic mean CT of four reference genes (*GAPDH, PGK1, POLR2A* and *RPL32*) was used for internal normalization (eΔCT) and corresponding gene expression levels (= 2^-eΔCT^) were divided by the average gene expression levels observed in donor samples.

***Mathematical model of burst-like transcription and cell-to-cell allelic imbalance***

We employed our previously published mathematical model to simulate the effects of burst-like transcription of *TNNI3* in single cells (1). We adapted input parameters of the model to best-fit bursting kinetics of *TNNI3* gene, RNA processing and protein translation. We used the ploidy distribution of donor cardiomyocytes as previously determined^17^ (19.3% 2n, 45.4% 4n, 23.1% 8n, 11.3% 16n and 0.9% 32n). As small numbers of aTS were determined in the RNA-FISH experiments, activation and inactivation rate constant of transcription was adjusted to short transcriptional bursts to match the aTS distribution observed experimentally. The inactivation rate constant specifies the mean number of transitions of one allele from active to inactive state per unit of time. It was adjusted to 1.2 transitions/h for both alleles. The activation rate constant specifies the mean number of transitions of one allele from inactive to active state and was adjusted to 0.018 transitions/h for both alleles. The reciprocal of the rate constants represent the mean active and inactive time of each allele (50 min and nearly 56 h, respectively). Therefore, each allele is inactive for 98.5% of the time. Pre-mRNA synthesis rate for both alleles was set to 192 molecules/h to match the mean *TNNI3*-mRNA count per single cell as determined experimentally. The mRNA synthesis rate represents the splicing rate of pre-mRNA to mRNA. For *TNNI3* it was adjusted to 0.9 molecules/h. In mouse embryonic stem cells, degradation of *TNNI3-*mRNA was seen to be rather slow with a rate constant of 0.03 molecules/h(14), which was used as an approach for human cardiomyocytes. Protein synthesis rate constant was set to 6,000 molecules per hour and per mRNA molecule to meet the estimated mean cTnI abundance in cardiomyocytes. Protein degradation rate constant of cTnI was taken from the literature with 0.009 molecules/h for both alleles (15). The simulation run was performed for 100,000,000 simulated cells, resulting in robust value distributions. We calculated the simulated distribution of exerted force at submaximal activation at pCa 5.24 from the simulated fraction of mutant cTnI. The calculation was based on the assumption that higher fractions of mutant protein produce a proportionally higher effect on force development. As the mutation R145W in cTnI causes a reduction of force on average, we associated low forces with high fractions of mutant protein. Variance of donor force was accepted as experimental error and added to the variance of simulated mutant protein fractions as described previously (1). To match the effect of the mutation on force reduction, median of simulated fractions was set to median of patient’s mutant protein fractions, after logit transformation.

The simulated force distribution resembles patient’s force distribution to a high degree and is markedly different from donor’s force distribution. This supports the hypothesis that mRNA-allelic imbalance can lead to unequal fraction of mutant and WT-protein and subsequently to contractile imbalance.

**Supplementary References**

1. Montag J, Kowalski K, Makul M, Ernstberger P, Radocaj A, Beck J, Becker E, Tripathi S, Keyser B, Mühlfeld C, et al. Burst-like transcription of mutant and wildtype MYH7-alleles as possible origin of cell-to-cell contractile imbalance in Hypertrophic Cardiomyopathy. *Front Physiol* (2018) 9:359. doi: 10.3389/fphys.2018.00359

2. Lyubimova A, Itzkovitz S, Junker JP, Fan ZP, Wu X, Van Oudenaarden A. Single-molecule mRNA detection and counting in mammalian tissue. *Nat Protoc* (2013) 8:1743–1758. doi: 10.1038/nprot.2013.109

3. Levesque MJ, Raj A. Single-chromosome transcriptional profiling reveals chromosomal gene expression regulation. *Nat Methods* (2013) 10:246–248. doi: 10.1038/nmeth.2372

4. Haase A, Göhring G, Martin U. Generation of non-transgenic iPS cells from human cord blood CD34+ cells under animal component-free conditions. *Stem Cell Res* (2017) 21:71–73. doi: 10.1016/j.scr.2017.03.022

5. Weber N, Kowalski K, Holler T, Radocaj A, Fischer M, Thiemann S, de la Roche J, Schwanke K, Piep B, Peschel N, et al. Advanced Single-Cell Mapping Reveals that in hESC Cardiomyocytes Contraction Kinetics and Action Potential Are Independent of Myosin Isoform. *Stem Cell Reports* (2020) 14:788–802. doi: 10.1016/j.stemcr.2020.03.015

6. Boon WC, Petkovic-duran K, White K, Tucker E, Albiston A, Horne MK, Aumann TD. Acoustic microstreaming increases the efficiency of reverse transcription reactions comprising single-cell quantities of RNA. *Biotechniques* (2011) 50:116–119. doi: 10.2144/000113587

7. Olivetti G, Cigola E, Maestri R, Corradi D, Lagrasta C, Gambert SR, Anversa P. Aging, Cardiac Hypertrophy and Ischemic Cardiomyopathy Do Not Affect the Proportion of Mononucleated and Multinucleated Myocytes in the Human Heart. *J Mol Cell Cardiol* (1996) 28:1463–1477. doi: 10.1006/jmcc.1996.0137.

8. Tripathi S, Schultz I, Becker E, Montag J, Borchert B, Francino A, Navarro-Lopez F, Perrot A, Özcelik C, Osterziel KJ, et al. Unequal allelic expression of wild-type and mutated β-myosin in familial hypertrophic cardiomyopathy. *Basic Res Cardiol* (2011) 106:1041–1055. doi: 10.1007/s00395-011-0205-9

9. Thompson JR, Marcelino LA, Polz MF. Heteroduplexes in mixed-template amplifications: formation, consequence and elimination by “reconditioning PCR.” *Nucleic Acids Res* (2002) 30:2083–2088. doi: 10.1093/nar/30.9.2083

10. Kraft T, Witjas-Paalberends ER, Boontje NM, Tripathi S, Brandis A, Montag J, Hodgkinson JL, Francino A, Navarro-lopez F, Brenner B, et al. Familial hypertrophic cardiomyopathy: Functional effects of myosin mutation R723G in cardiomyocytes. *J Mol Cell Cardiol* (2013) 57:13–22. doi: 10.1016/j.yjmcc.2013.01.001

11. Föhr KJ, Warchol W, Gratzl M. Calculation and Control of Free Divalent Cations in Solutions Used for Membrane Fusion Studies. *Methods Enzymol* (1993) 221:149–157. doi: 10.1016/0076-6879(93)21014-y

12. Ye J, Coulouris G, Zaretskaya I, Cutcutache I, Rozen S, Madden TL. Primer-BLAST: A tool to design target-specific primers for polymerase chain reaction. *BMC Bioinformatics* (2012) 13:1–11.

13. Riedel G, Rüdrich U, Fekete-Drimusz N, Manns MP, Vondran FWR, Bock M. An extended ΔCT-method facilitating normalisation with multiple reference genes suited for quantitative RT-PCR analyses of human hepatocyte-like cells. *PLoS One* (2014) 9:2–6. doi: 10.1371/journal.pone.0093031

14. Sharova L V., Sharov AA, Nedorezov T, Piao Y, Shaik N, Ko MSH. Database for mRNA Half-Life of 19 977 Genes Obtained by DNA Microarray Analysis of Pluripotent and Differentiating Mouse Embryonic Stem Cells. *DNA Res* (2009) 16:45–58. doi: 10.1093/dnares/dsn030

15. Martin AF. Turnover of cardiac troponin subunits. Kinetic evidence for a precursor pool of troponin-I. *J Biol Chem* (1981) 256:964–968. doi: 10.1017/S1431927612003613
